# Inter‐Atomic Synergy on Single‐Atom Alloy Promotes Cyclohexanone Oxime Electrosynthesis

**DOI:** 10.1002/adma.72807

**Published:** 2026-03-17

**Authors:** Panlong Zhai, Chen Wang, Guan Sheng, Chao Ye, Jungang Hou, Qinfen Gu, Tao Ling, Ye Zhu, Pei Liang, Xin Wang, Jieqiong Shan

**Affiliations:** ^1^ Department of Chemistry City University of Hong Kong Kowloon Hong Kong SAR P. R. China; ^2^ State Key Laboratory of Fine Chemical School of Chemical Engineering Dalian University of Technology Dalian P. R. China; ^3^ Department of Applied Physics Research Institute for Smart Energy The Hong Kong Polytechnic University Kowloon Hong Kong SAR P. R. China; ^4^ School of Chemical Engineering The University of Adelaide Adelaide South Australia Australia; ^5^ Australian Synchrotron ANSTO Clayton Australia; ^6^ School of Materials Science and Engineering Tianjin University Tianjin P. R. China; ^7^ College of Optical and Electronic Technology China Jiliang University Hangzhou P. R. China; ^8^ Shenzhen Research Institute City University of Hong Kong Shenzhen P. R. China

**Keywords:** atomic‐scale synergistic mechanism, cyclohexanone oxime, electrocatalytic C─N coupling reaction, single‐atom alloy

## Abstract

The electrosynthesis of cyclohexanone oxime from cyclohexanone and nitrogenous feedstock driven by renewable electricity presents a sustainable alternative to energy‐intensive and hazardous industrial processes. However, achieving high activity and selectivity is challenged by the over‐reduction of key intermediates and the lack of effective sites for C─N coupling. Herein, we report a Fe_1_Bi single‐atom alloy (Fe_1_Bi SAA) featuring Fe‐Bi atomic interfaces that collaborate for the one‐pot electrosynthesis of cyclohexanone oxime. The Fe_1_Bi SAA achieves a remarkable Faradaic efficiency of 70.9% and a yield rate of 0.94 mmol cm^−2^ h^−1^ for cyclohexanone oxime. Combined in situ electrochemical spectroscopic measurements and density functional theory calculations reveal an atomic‐scale synergistic mechanism: dispersed Fe sites adsorb and activate cyclohexanone, while adjacent Bi sites selectively reduce nitrite to the key hydroxylamine intermediate. The techno‐economic analysis based on flow electrolyzer operation confirms the potential economic viability of the electrosynthesis of cyclohexanone oxime. This work provides profound atomic‐level insight into cooperative catalysis for C─N coupling reactions toward the electrosynthesis of value‐added organonitrogen compounds.

## Graphical abstract

Electrosynthesis of cyclohexanone oxime using renewable electricity offers a greener alternative to traditional energy‐intensive methods. Here, we develop a Fe_1_Bi single‐atom alloy that delivers 70.9% Faradaic efficiency and 0.94 mmol cm^−2^ h^−1^ yield rate through the atomic‐scale synergistic mechanism. The techno‐economic analysis confirms the economic viability of the electrosynthesis of cyclohexanone oxime.

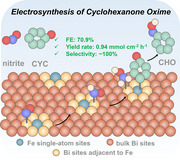

## 1 Introduction

**FIGURE 1:**
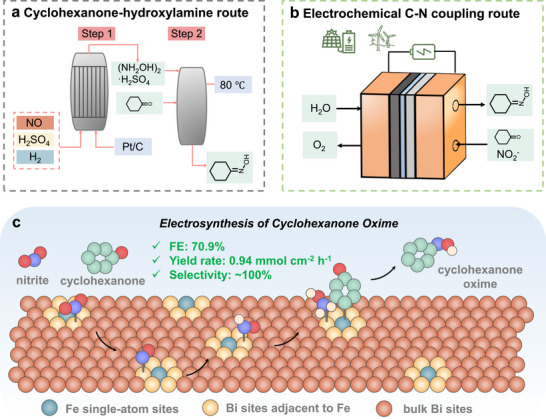
Comparison of (a) conventional cyclohexanone‐hydroxylamine route and (b) electrochemical C─N coupling route for the synthesis of cyclohexanone oxime. (c) Schematic diagram of cyclohexanone oxime electrosynthesis over Fe_1_Bi single‐atom alloy catalyst.

Organonitrogen compounds are ubiquitous in modern agriculture, biomedicine, and manufacturing. Among these, cyclohexanone oxime (CHO) serves as a vital feedstock for producing ε‐caprolactam, the primary monomer for Nylon‐6, which is widely utilized in textiles, automotive components, and medical apparatus [1]. With Nylon‐6 production projected to reach 8.9 million tonnes annually, the demand for efficient and sustainable cyclohexanone oxime synthesis is steadily increasing [2]. Industrially, production relies predominantly on two routes: the cyclohexanone‐hydroxylamine process or the cyclohexanone ammoximation process (Figure 1a; Figure S1) [3, 4, 5, 6]. The former route necessitates production of hydroxylamine using explosive hydrogen and corrosive SO_2_ and NO_x_ over noble metal catalysts, raising significant safety, cost, and environmental concerns, while generating large quantities of low‐value ammonium sulfate by‐products [7]. While the ammoximation route employing ammonia and hydrogen peroxide (H_2_O_2_) over a titanium silicate‐1 (TS‐1) catalyst avoids some hazards, its dependence on H_2_O_2_ synthesized via the energy‐intensive anthraquinone process imposes substantial economic and logistical burdens related to transportation and storage [8]. Consequently, developing a mild, safe, and sustainable alternative for large‐scale cyclohexanone oxime production remains an urgent imperative.

Electrosynthesis offers a promising pathway for synthesizing value‐added organonitrogen compounds under mild reaction conditions using renewable electricity, minimizing environmental impact and safety risks [9, 10, 11, 12, 13]. A particularly attractive strategy involves the electrocatalytic reduction of nitrogen oxides (NO_x_), utilizing water as the proton source to generate hydroxylamine intermediates (^*^NH_2_OH) in situ. This reactive nucleophilic reagent can then spontaneously couple with carbonyl compounds (aldehydes/ketones) to form oximes (Figure 1b) [14, 15]. However, the efficient electrosynthesis of oximes through this route faces a fundamental challenge: the multi‐step proton/electron transfer required for ^*^NH_2_OH generation is highly susceptible to over‐reduction, leading predominantly to ammonia formation, rather than desired C─N coupling with ketones [16, 17, 18].

Thus, the rational design of electrocatalysts capable of selectively generating the ^*^NH_2_OH intermediate and facilitating its subsequent coupling with carbonyl species is paramount. P‐block metals, particularly bismuth, typically exhibit moderate nitrogen binding energies and weak hydrogen adsorption [19], inherently catalyzing NO_x_ reduction to NH_2_OH with high Faradaic efficiency (FE) while suppressing the competing hydrogen evolution reaction (HER) [20, 21]. However, their limited ability to provide diverse active sites hinders efficient adsorption and activation of the organic carbonyl reactants (e.g., cyclohexanone, CYC), thereby impeding the crucial C─N coupling step [22]. Conversely, hybridizing with d‐block metal can enhance the C─N coupling interactions by providing multiple active sites but risk activating HER and destabilizing ^*^NH_2_OH intermediate [23, 24]. Single‐atom alloy (SAA) catalysts, featuring atomically dispersed guest metals within a host metal matrix, present an ideal platform to overcome these limitations [25, 26, 27, 28, 29, 30]. The isolated guest atoms can electronically disturb the neighboring host sites, optimizing adsorption energies for key intermediates like ^*^NH_2_OH and ^*^CYC, while the distinct atomic sites offer the potential for synergistic cooperation in multi‐step reactions [31, 32]. Despite this promise, the deliberate design and mechanistic understanding of SAA catalysts for facilitating challenging electrocatalytic C─N coupling reactions remain largely unexplored.

Herein, we report the targeted design and synthesis of Fe_1_Bi SAA electrocatalyst for the electrosynthesis of cyclohexanone oxime from nitrite and cyclohexanone (Figure 1c). The optimized Fe_1_Bi SAA delivers excellent performance, achieving a high cyclohexanone oxime FE of 61.3% and a remarkable yield rate of 0.27 mmol cm^−2^ h^−1^ in an H‐cell, with nearly‐100% ketone conversion and oxime selectivity. Critically, we achieve a high FE of 70.9% and a yield rate of 0.94 mmol cm^−2^ h^−1^ in a membrane electrode assembly (MEA) electrolyzer. Through the combination of in situ characterization and density functional theory (DFT) calculations, we unveil the atomic‐scale synergistic mechanism: atomically dispersed Fe sites alter the electronic structure of adjacent Bi sites to promote the selective reduction of NO_2_^−^ to the key ^*^NH_2_OH intermediate, simultaneously, the Fe sites efficiently adsorb and activate cyclohexanone, which enhances the critical C─N coupling step in the cyclohexanone oxime electrosynthesis. The unique Fe‐Bi atomic interface facilitates C─N coupling by optimizing the adsorption configuration and energy landscape of both critical intermediates. The techno‐economic analysis based on MEA operation over Fe_1_Bi SAA confirms the economic viability potential of the oxime electrosynthesis. This work not only demonstrates a highly efficient and sustainable route to a critical chemical feedstock but also provides fundamental atomic‐level insight into designing cooperative active sites for complex electrochemical C─N bond formation.

## 2 Results and Discussion

### 2.1 Design and Characterization of Electrocatalysts

**FIGURE 2:**
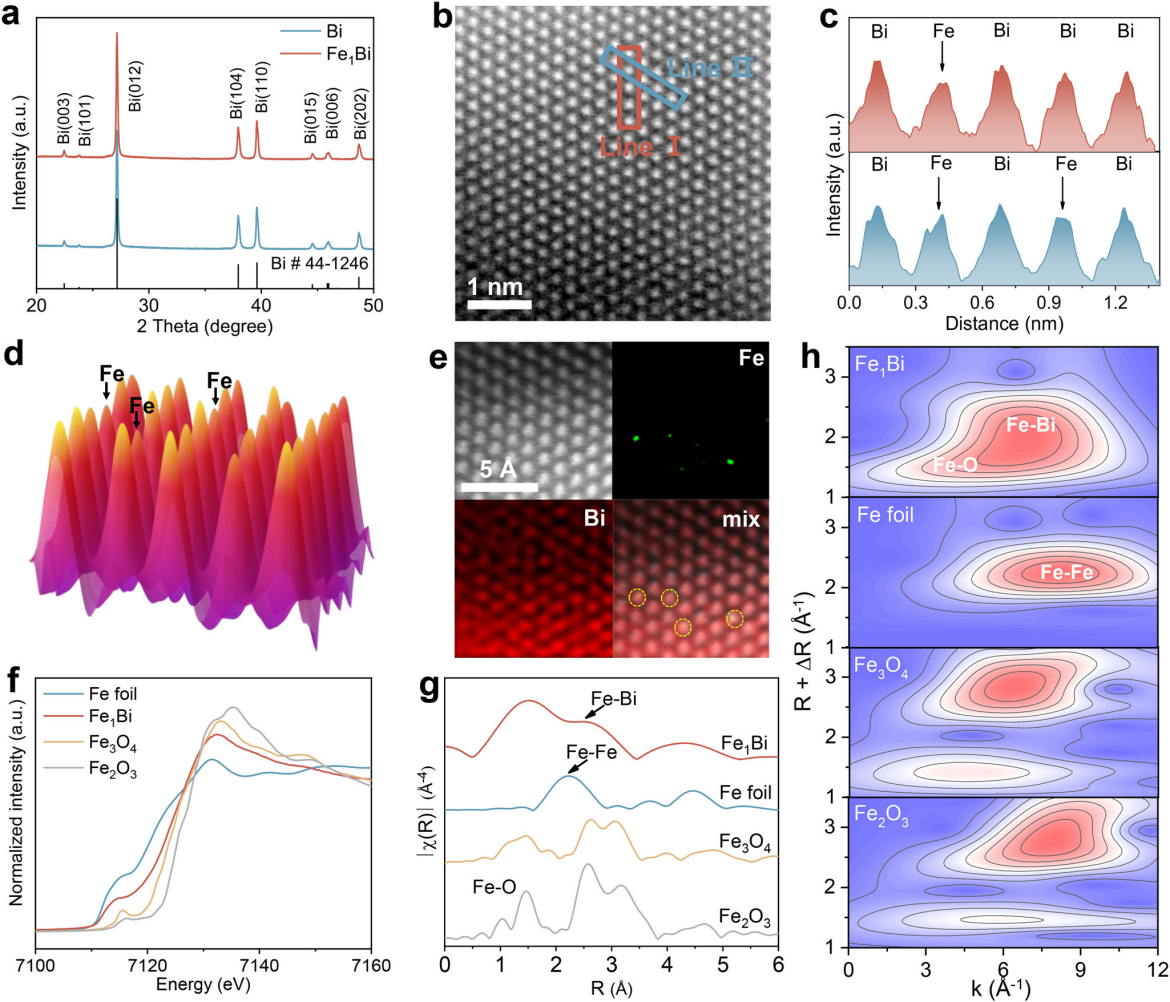
(a) XRD patterns of pristine Bi and Fe_1_Bi SAA. (b) Aberration‐corrected HAADF‐STEM image of Fe_1_Bi SAA. (c) Atom‐column intensity profiles along the marked lines. (d) 3D topographic atom‐column intensity map. (e) Atomically resolved HAADF‐STEM image and corresponding EDS elemental maps of Fe_1_Bi SAA. (f) Fe K‐edge XANES spectra, (g) Fourier‐transformed EXAFS spectra, and (h) wavelet transform‐EXAFS of Fe_1_Bi SAA and the reference.

We designed Fe_1_Bi SAA electrocatalysts with different Fe loadings via co‐reduction synthesis, with pristine Bi synthesized comparably [33]. Inductively coupled plasma‐atomic emission spectrometry (ICP‐AES) measurement reveals that the optimal Fe content in Fe_1_Bi SAA is 0.99 wt.%. The X‐ray diffraction (XRD) patterns of both Fe_1_Bi SAA and pristine Bi display characteristic diffraction peaks of metallic Bi (JCPDS #44‐1246), with no peaks attributed to metallic Fe or Fe oxides, indicating the atomic dispersion of Fe atoms within the Fe_1_Bi SAA (Figure 2a) [34]. Raman spectroscopy further verifies the structural integrity of Fe_1_Bi SAA, showing similar E_g_ and A_1g_ stretching modes of metallic Bi─Bi bonds at 72 and 98 cm^−1^ in both Fe_1_Bi SAA and pristine Bi (Figure S2).

Morphological studies by field emission‐scanning electron microscopy (FE‐SEM) and transmission electron microscopy (TEM) reveal a spherical agglomerated shape for both pristine Bi and Fe_1_Bi SAA, confirming that Fe incorporation does not perturb the Bi framework (Figures S3–S5). The high‐resolution TEM (HR‐TEM) image reveals a distinct lattice space of 0.327 nm for Fe_1_Bi SAA, which can be indexed as the Bi (012) plane, consistent with the XRD results (Figure S5d) [21, 35]. Furthermore, the atomic structure of Fe_1_Bi SAA was investigated by aberration‐corrected high‐angle annular dark‐field scanning transmission electron microscopy (AC HAADF‐STEM). While the low Z‐contrast of Fe relative to Bi poses the direct observation of Fe atoms as challenging (Figure 2b; Figure S6), the presence of lower‐intensity peaks in the atomic intensity profile along lines I and II in marked rectangles indicates the presence of atomically dispersed Fe in the Bi matrix (Figure 2c) [36]. A 3D atomic‐column intensity map further identifies the isolated Fe atoms in Fe_1_Bi SAA (Figure 2d). The lower‐intensity peaks correspond to Fe atoms, which are dispersed by occupying the host Bi positions rather than occupying interstitial sites within the metallic Bi lattice, eliminating ambiguity regarding atomic dispersion of Fe [37]. Moreover, an atomically resolved HAADF‐STEM image and corresponding energy‐dispersive X‐ray spectroscopy (EDS) elemental mapping verified the uniform distribution of Fe throughout the Bi matrix (Figure 2e; Figure S7).

The electronic structure and coordination environment of Fe_1_Bi SAA and Bi were investigated by X‐ray absorption fine structure (XAFS) spectroscopy. The Fe K‐edge X‐ray absorption near‐edge structure (XANES) spectrum of Fe_1_Bi SAA exhibits an absorption edge between those of Fe foil and Fe_2_O_3_ (Figure 2f). Critically, the Fourier‐transformed extended X‐ray absorption fine structure (FT‐EXAFS) spectra of Fe_1_Bi SAA show a primary peak at 1.5 Å corresponding to Fe─O coordination, and a definitive second‐shell peak at 2.5 Å that can be exclusively assigned to Fe─Bi coordination (Figure 2g). We do not observe Fe─Fe coordination in the Fe K‐edge EXAFS spectra of Fe_1_Bi SAA. These results indicate that Fe species are atomically dispersed within the Bi matrix, forming atomically adjacent Fe─Bi dual atomic sites [27]. Wavelet transform EXAFS (WT‐EXAFS) further distinguished the Fe─Bi scattering path in Fe_1_Bi SAA with an intensity maximum at 6.9 Å^−1^ from the Fe─Fe path of Fe foil at 8.4 Å^−1^, excluding the Fe clustering (Figure 2h) [26]. Quantitative EXAFS fitting results (Figure S8 and Table S1) show that the average Fe─O and Fe─Bi coordination numbers in Fe_1_Bi SAA are 6.4 and 4.2, respectively, consistent with Fe atoms occupying Bi lattice sites surrounded by approximately four adjacent Bi atoms. The Bi L_III_‐edge XANES and corresponding FT‐EXAFS spectra reveal the metallic state of Bi with intralayer and interlayer Bi─Bi bonds in Fe_1_Bi SAA; while a subtle negative energy shift versus pristine Bi indicates electron transfer from Fe to Bi (Figure S9) [38, 39]. The electronic perturbation in Fe_1_Bi SAA can be further evidenced by Bi 4f X‐ray photoelectron spectroscopy (XPS) spectra, which show a 0.1 eV negative shift of Bi binding energy in comparison with pristine Bi (Figure S10) [40]. Collectively, these results support the structural identification of Fe_1_Bi SAA—the substitutional Fe incorporation induces Fe‐Bi interaction via electron transfer while maintaining the Bi host framework.

### 2.2 Electrocatalytic C─N Coupling Reaction Evaluation

**FIGURE 3:**
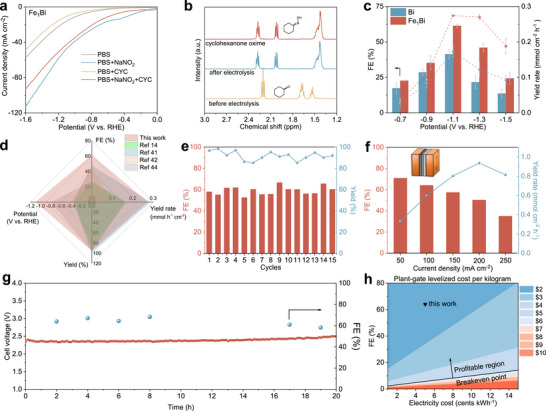
(a) LSV curves of Fe_1_Bi SAA in different electrolytes. (b) ^1^H NMR spectra of cyclohexanone oxime and cyclohexanone. (c) Potential‐dependent FE and yield rate of cyclohexanone oxime for Fe_1_Bi SAA and pristine Bi. (d) Comparison of the FE, yield rate, yield, and applied potential of Fe_1_Bi SAA in H‐cell with the corresponding performance metrics from representative reports. (e) Stability test of Fe_1_Bi SAA at ‐1.1 V vs. RHE. (f) FE of cyclohexanone oxime for Fe_1_Bi SAA in the flow electrolyzer. (g) Durability test of the MEA at a current density of 100 mA cm^−2^. (h) Plant‐gate levelized cost of cyclohexanone oxime from TEA.

The electrocatalytic performance of Fe_1_Bi SAA for cyclohexanone oxime electrosynthesis was evaluated in an H‐type electrochemical cell using a three‐electrode configuration. To preserve the chemical stability of the nitrogen source and oxime product, all reactions were conducted in a neutral phosphate‐buffered solution (PBS) electrolyte (Figure S11) [27]. As shown in linear sweep voltammetry (LSV) curves, Fe_1_Bi SAA exhibits significantly increased current density upon the addition of nitrite into PBS electrolyte, suggesting its efficient catalytic activity for nitrite reduction (NO_2_RR). A slight decrease is observed upon the addition of cyclohexanone, suggesting that the C─N coupling may be involved in the reaction (Figure 3a; Figure S12). Subsequently, chronoamperometry measurements were performed at different potentials to systematically assess the performance of the electrocatalytic C─N coupling reaction. Organic compounds were quantified using gas chromatography‐mass spectrometry (GC‐MS) and ^1^H nuclear magnetic resonance spectroscopy (^1^H NMR) [14, 15], and NH_4_^+^ was quantified by colorimetric methods through ultraviolet–visible (UV–vis) spectrophotometer (Figures S13–S16). The ^1^H NMR spectra reveal that the peak of cyclohexanone disappears, accompanied by the emergence of characteristic peaks corresponding to cyclohexanone oxime, indicating cyclohexanone oxime as the exclusive organic product (Figure 3b; Figure S17a). The chemical shifts at 160.6, 32.0, 26.9, 25.8, 25.7, and 24.5 ppm in the ^13^C NMR spectrum are assigned to cyclohexanone oxime (Figure S17b) [14]. Additionally, the characteristic molecular ion peak with mass to charge (m/z) of 113.1 from GC‐MS is identical to the molecular weight of cyclohexanone oxime, further confirming the successful synthesis of cyclohexanone oxime (Figure S17c). The FE and yield rate for cyclohexanone oxime on Fe_1_Bi SAA show a volcano‐type trend, reaching up to 61.3% and 0.27 mmol cm^−2^ h^−1^ at ‐1.1 V vs. RHE, respectively. A multi‐parameter radar plot comparison under similar near‐neutral electrolyte conditions demonstrates the overall superior performance profile of Fe_1_Bi SAA against previously reported catalysts (Figure 3c,d) [14, 41, 42, 43, 44]. The yield of cyclohexanone oxime is 95.3% at ‐1.1 V vs. RHE, revealing that the conversion of cyclohexanone is nearly complete (Figure S18). In contrast, pristine Bi catalyst exhibits much lower FE and yield rate of 41.4% and 0.17 mmol cm^−2^ h^−1^, respectively. The decrease in FE of cyclohexanone oxime at more negative potential is attributed to the enhanced competition from NO_2_^−^ over‐reduction to NH_4_^+^ and N_2_, as well as the HER pathways that are significantly suppressed on the Fe_1_Bi SAA (Figure S19).

The temporal concentration variation of the reactant is monitored, showing that cyclohexanone is nearly completely depleted after 2500 s (Figure S20). The yield of cyclohexanone oxime exhibits an inverse trend relative to cyclohexanone, manifesting carbon selectivity close to 100%. Moreover, the effect of Fe loading in Fe_1_Bi SAA is explored by studying similar electrocatalysts with lower (0.55 wt.%, FeBi‐1) and higher (1.85 wt.%, FeBi‐2) Fe contents (Figure S21). Notably, both catalysts demonstrate lower FE and yield rate toward cyclohexanone oxime in comparison with Fe_1_Bi SAA, suggesting the critical role of Fe species with optimal loadings for cyclohexanone oxime electrosynthesis. Lower Fe loading may provide insufficient active sites for the adsorption of reactant or key intermediates, whereas excessive Fe loading can induce aggregation of Fe sites, thereby promoting the competing HER (Figures S22 and S23). Moreover, to balance the tradeoff between FE and yield, an optimal cyclohexanone‐to‐nitrite molar ratio of 10 is determined and utilized in the synthesis of cyclohexanone oxime (Figure S24).

In addition, the Fe_1_Bi SAA exhibits a higher intrinsic activity in comparison with pristine Bi when normalized to electrochemically active surface area (Figure S25). This can be attributed to the superior interfacial charge transfer kinetics of Fe_1_Bi SAA, as demonstrated by a smaller semi‐circle diameter in electrochemical impedance spectroscopy (EIS) measurements (Figure S26). Importantly, the Fe_1_Bi SAA demonstrates good electrochemical durability over 15‐cycle continuous electrolysis, which achieves stable FE and yield of over 60% and 90%, respectively, with slight fluctuations (Figure 3e). According to XRD, XPS, and HR‐TEM results, the Fe_1_Bi SAA remains unchanged after cyclic electrolysis, indicating its high structural stability during electrocatalysis (Figure S27). Moreover, the electrocatalytic system shows broad substrate generality, enabling the efficient conversion of various aldehydes and ketones to their corresponding oximes with high FEs (Figure S28), thereby highlighting the versatility of the Fe_1_Bi SAA catalyst.

The MEA flow reactor with a two‐electrode configuration was assembled to assess the practical application potential of oxime electrosynthesis via electrocatalytic C─N coupling. The Fe_1_Bi SAA and NiFe layer double hydroxide serve as cathode and anode, respectively, and are separated by a proton exchange membrane. On the cathode side, a continuous flow of a mixture consisting of PBS, nitrite, and cyclohexanone is supplied by a peristaltic pump. Besides, the cyclohexanone oxime collected from the cathode effluent was quantified to evaluate the performance of oxime electrosynthesis. Chronopotentiometry tests were conducted at current densities ranging from 50 to 250 mA cm^−2^, achieving FE of 70.9% at 50 mA cm^−2^ (Figure 3f). The maximum yield rate of cyclohexanone oxime for Fe_1_Bi SAA reaches 0.94 mmol cm^−2^ h^−1^ at 200 mA cm^−2^ with the FE of 50.2%, surpassing most of the previously reported performance in similar electrocatalytic systems (Table S2) [14, 23, 41, 42, 43, 44, 45]. Then, the stability of the MEA at 100 mA cm^−2^ was evaluated. The FE and cell voltage remain stable within 20 h (Figure 3g). Furthermore, a preliminary techno‐economic analysis (TEA) on plant‐gate levelized cost was performed to evaluate the industrial application potential and economic viability of the Fe_1_Bi SAA‐based cyclohexanone oxime electrosynthesis approach powered by renewable electricity. The capital, operating, and raw material costs are mainly considered. The current density and FE influence the capital costs, while the electricity cost affects the operating cost [46, 47]. Single‐variable sensitivity analysis is performed to determine the cost‐relevant parameters for cyclohexanone oxime electrosynthesis, revealing that the plant‐gate levelized cost is mainly influenced by FE, electricity cost, and nitrite cost (Figure S29). At the electricity price of 5 cents kWh^−1^ and the optimal electrocatalytic conditions (100 mA cm^−2^, 2.4 V, 64.0%) in the flow electrolyzer, the C─N coupling reaction to synthesize cyclohexanone oxime surpassed the profitable threshold, highlighting the promising application of cyclohexanone oxime electrosynthesis (Figure 3h).

### 2.3 In Situ Spectroscopic Analysis

**FIGURE 4:**
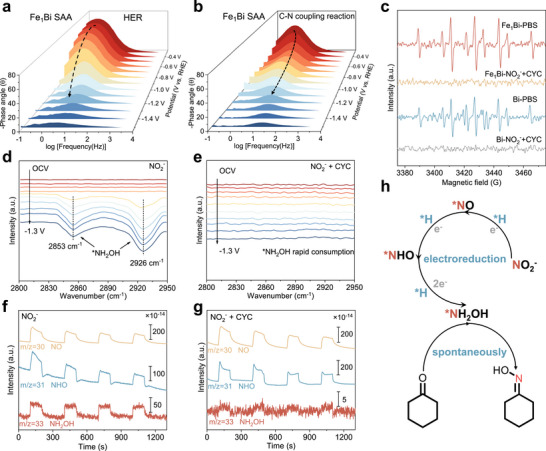
Bode plots of Fe_1_Bi SAA at different potentials for (a) HER and (b) C─N coupling reaction. (c) DMPO‐involved EPR spectra of the Fe_1_Bi SAA and pristine Bi under different electrolysis conditions. In situ ATR‐SEIRAS spectra of Fe_1_Bi SAA at different potentials for (d) NO_2_RR and (e) C─N coupling reaction. In situ DEMS of Fe_1_Bi SAA for (f) NO_2_RR and (g) C─N coupling reaction. (h) Schematic illustration of the cyclohexanone oxime generation pathway.

To elucidate the origin of the high FE for cyclohexanone oxime electrosynthesis on Fe_1_Bi SAA, we conducted a series of operando investigations to clarify the C─N coupling pathway. Operando EIS at different potentials revealed distinct reaction kinetics for C─N coupling reaction versus the competing protonation (HER) pathway. The equivalent circuit modeling identified four key components (Figure S30): electron transfer from Fe_1_Bi SAA to the reaction interface (R_1_), intermediate accumulation (R_2_), the charge transfer during interfacial reaction (R_3_), and electrolyte resistance (R_4_) [48, 49]. In the Bode plot, changes in intensity and phase angle reflect interfacial charge transfer kinetics and accumulation/consumption of reaction intermediates, respectively (Figure 4a,b). In blank PBS without nitrite and cyclohexanone, the low‐frequency phase‐angle feature of Fe_1_Bi SAA indicates sluggish formation of adsorbed hydrogen (^*^H) via the Volmer step; as the potential becomes more negative, the phase‐angle peak gradually shifts toward the middle‐frequency region, consistent with enhanced consumption of ^*^H via the Heyrovsky step during HER. In sharp contrast, after introducing nitrite and cyclohexanone, the phase‐angle peak shifts much more rapidly from low to middle frequencies, accompanied by a pronounced decrease in phase‐angle intensity as the potential decreases, indicating accelerated kinetics for ^*^H generation and rapid consumption of ^*^H by the C─N coupling sequence rather than H_2_ evolution; this interpretation is further supported by the smaller charge‐transfer resistance under C─N coupling conditions (Figure S31). Additionally, CV curves corroborate these observations by measuring the oxidation peak at ‐0.2 V vs. RHE, which can be assigned to ^*^H [50]. The ^*^H peak area of Fe_1_Bi SAA is greater than that of pristine Bi, confirming enhanced ^*^H generation (Figure S32). To obtain direct spectroscopic evidence for ^*^H generation, we performed electron paramagnetic resonance (EPR) measurements using 5,5‐dimethyl‐1‐pyrroline‐N‐oxide (DMPO) as a spin‐trapping agent. A clear DMPO‐H adduct signal was observed in 0.5 m PBS with the characteristic multi‐line pattern with the intensity ratio 1:1:2:1:2:1:2:1:1, confirming the ^*^H formation (Figure 4c) [50]. Notably, the DMPO‐H signal intensity for Fe_1_Bi SAA is markedly stronger than that of pristine Bi, demonstrating its superior ^*^H generation activity. Furthermore, the DMPO‐H signal vanished upon the addition of nitrite and cyclohexanone, indicating the rapid consumption of ^*^H species during the NO_2_RR.

To elucidate the reaction mechanism of cyclohexanone oxime electrosynthesis, we conducted in situ electrochemical attenuated total reflectance surface‐enhanced infrared absorption spectroscopy (ATR‐SEIRAS) to achieve the direct observation of key reaction intermediates. As shown in Figure 4d, during NO_2_RR, the N─H stretching vibrations at 2853 and 2926 cm^−1^ corresponded to the formation of ^*^NH_2_OH [24, 43]. Upon introducing cyclohexanone, the intensity of these peaks decreases significantly, suggesting the rapid nucleophilic attack of cyclohexanone by ^*^NH_2_OH, generating cyclohexanone oxime via C─N coupling (Figure 4e). In stark contrast, the weak signals for ^*^NH_2_OH are observed on pristine Bi. However, after the introduction of cyclohexanone, the peaks remain prominent, suggesting that incorporation of Fe enhances the absorption of cyclohexanone and facilitates the C─N coupling reaction (Figure S33). Moreover, online differential electrochemical mass spectrometry (DEMS) was employed to capture the gaseous intermediates during NO_2_RR and C─N coupling reactions (Figure 4f,g). The signal at m/z values of 30, 31, and 33 can be assigned to ^*^NO, ^*^NHO, and ^*^NH_2_OH species, respectively, confirming the stepwise NO_2_^−^ reduction to NH_2_OH on the Fe_1_Bi SAA surface [15, 51]. Notably, adding cyclohexanone led to immediate attenuation of the ^*^NH_2_OH signal, suggesting spontaneous C─N coupling between in situ‐generated ^*^NH_2_OH and cyclohexanone to form cyclohexanone oxime. Furthermore, enhanced ^*^NO and ^*^NHO signals are observed with the addition of cyclohexanone, attributed to accelerated NO_2_RR kinetics by the rapid consumption of ^*^NH_2_OH.

The contribution of a single atom of Fe in improving the performance of C─N coupling reaction was elucidated through a poisoning experiment using ethylenediaminetetraacetic acid disodium (EDTA) as the complexing reagent to coordinate with the Fe atom. A significant decrease in the FE is observed for the Fe_1_Bi SAA after the introduction of EDTA, while only a slight decrease was noted for pristine Bi (Figure S34). This suggests that the promotion of FE may stem from the adsorption of cyclohexanone at the Fe sites. Systematic control experiments were conducted to establish the reaction mechanism of the C─N coupling reaction (Table S3). No cyclohexanone oxime is detected without cyclohexanone, nitrite, or applied potential. Then, NH_4_^+^ is used as an alternative N‐containing reactant, while the absence of cyclohexanone oxime product excludes the ammonium involvement as nitrogen source and indicates the key role of NH_2_OH. Furthermore, the cyclohexanone oxime can be produced spontaneously when mixing NH_2_OH and cyclohexanone in a solution under ambient conditions. Therefore, the integrated evidence unambiguously reveals the reaction pathway: the nitrite reduction first occurs through a sequential deoxidation and hydrogenation process (NO_2_^−^ → ^*^NO_2_ → ^*^NO → ^*^NHO → ^*^NH_2_OH) to generate ^*^NH_2_OH, the ^*^CYC is rapidly attacked by nucleophilic ^*^NH_2_OH to yield cyclohexanone oxime (Figure 4h).

### 2.4 Theoretical Calculations and Mechanistic Analysis

**FIGURE 5:**
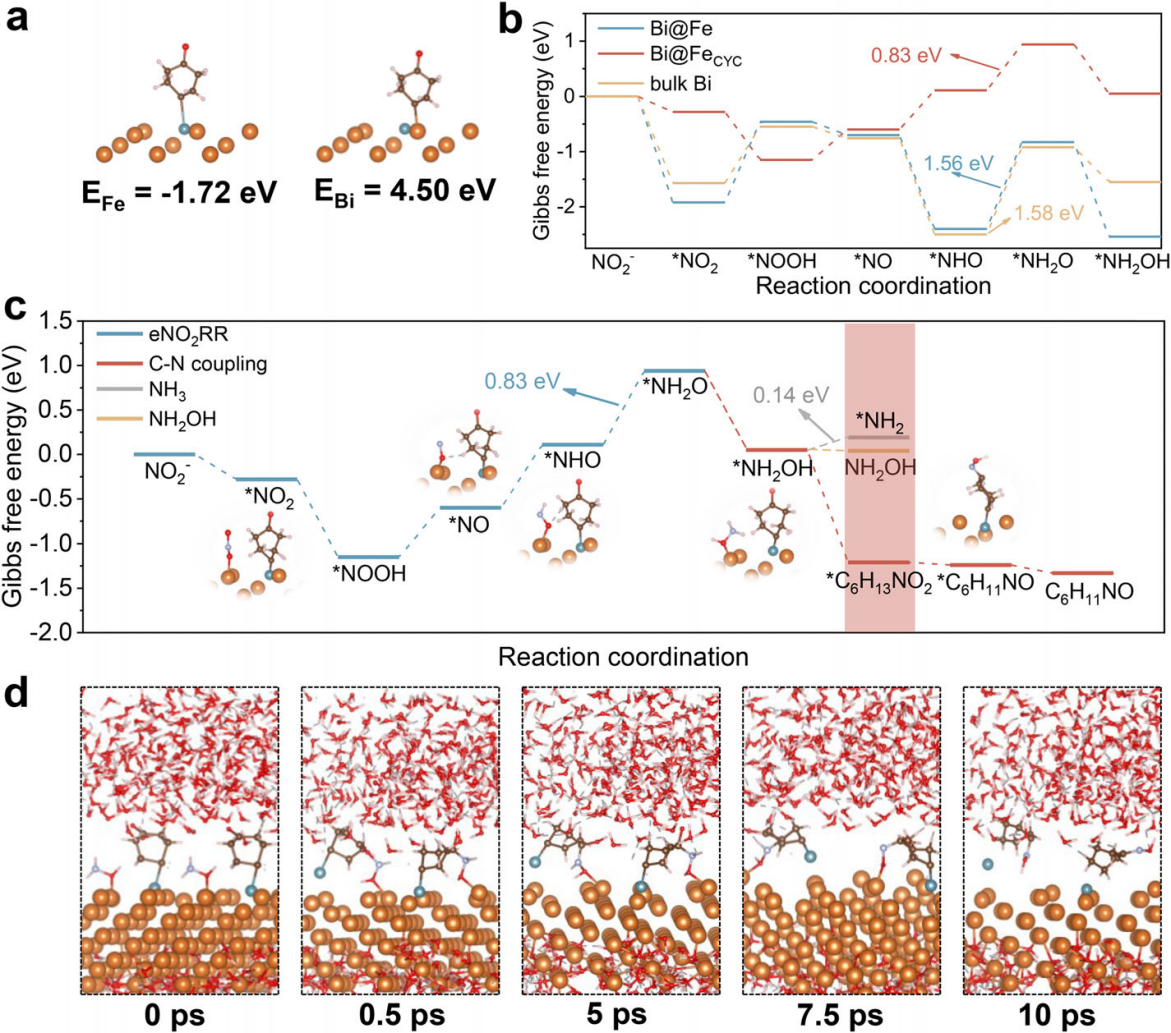
(a) The adsorption energies of cyclohexanone on the Fe and Bi sites of Fe_1_Bi SAA. The orange, blue, gray, brown, red, and white spheres represent Bi, Fe, N, C, O, and H, respectively. (b) Gibbs free energy profiles for nitrite reduction on different sites. (c) Gibbs free energy profiles for the co‐reduction of nitrite and cyclohexanone of Fe_1_Bi SAA. (d) The snapshots of the MD trajectories of the C─N coupling reaction at 0, 0.5, 5, 7.5, and 10 ps.

Complementing the in situ electrochemical spectroscopic evidence, DFT calculations were conducted to elucidate the origins of Fe_1_Bi SAA's exceptional performance and unveil the atomic‐scale mechanism of C─N coupling reaction [52]. The model of Bi(012) surface and atomically dispersed Fe on the surface of the Bi(012) surface were constructed to represent pristine Bi and Fe_1_Bi SAA, respectively, due to their lower formation energy (Figure S35). The calculated projected density of states (pDOS) analysis indicates the good electronic conductivity of both models with carriers crossing the Fermi level (Figure S36). The incorporation of Fe induces a downshift of the d‐band center from 0.81 eV of pristine Bi to 0.70 eV of Fe_1_Bi SAA, enhancing the adsorption strength of reaction intermediates. Furthermore, the differential charge density analysis illustrates that Fe sites serve as primary reactant adsorption sites (Figure S36c). As the initial key step in the C─N coupling reaction, the adsorption behavior of cyclohexanone was investigated. The atomically dispersed Fe sites exhibit a more negative adsorption energy (‐1.72 eV) compared to Bi sites (4.50 eV), indicating the preferential adsorption of cyclohexanone on Fe sites (Figure 5a). Free energy profiles for free energy changes (ΔG) of NO_2_RR to ^*^NH_2_OH were calculated across different sites (Figure 5b). On bulk Bi sites that are distant from Fe atoms, the ^*^NHO to ^*^NH_2_O step is the rate‐determining step (RDS) with an energy barrier of 1.58 eV. In contrast, the Bi sites adjacent to Fe atoms (Bi@Fe) exhibit the same RDS but lower the energy barrier to 1.56 eV. Critically, when ^*^CYC is pre‐adsorbed on a neighboring Fe site (Bi@Fe_CYC_), the ΔG for the RDS of ^*^NHO → ^*^NH_2_O drops dramatically to 0.83 eV, demonstrating a catalytic synergy where CYC adsorption promotes ^*^NH_2_OH formation. Furthermore, we also computed the alternative hydrogenation pathway from ^*^NHO to ^*^NHOH, which presents a higher energy barrier of 0.96 eV, and was also considered (Figure S37). In comparison, the formation of ^*^NH_2_O is energetically more favorable. This presents a significant improvement over pristine Bi with an RDS barrier of 1.87 eV (Figure S38).

The C─N coupling reaction pathway was mapped with comprehensive free energy profiles (Figure 5c; Figure S39). The critical intermediate for cyclohexanone oxime is ^*^C_6_H_13_NO_2_, which can be formed by nucleophilic attack of ^*^CYC on Fe by the ^*^NH_2_OH on adjacent Bi sites. The C─N coupling is a spontaneous step with an exergonic barrier of ‐1.26 eV. Compared to the desorption of ^*^NH_2_OH and over‐reduction to ^*^NH_2_, the C─N coupling reaction is energetically more favorable, aligning with the experimental observation of spontaneous reaction between cyclohexanone and NH_2_OH. The subsequent dehydration of ^*^C_6_H_13_NO_2_ to complete the synthesis of ^*^CHO (‐0.03 eV), along with the product desorption of ^*^CHO (‐0.09 eV), are both thermodynamically downhill. Moreover, ab initio molecular dynamics (AIMD) simulations were conducted to capture the dynamic trajectories of the cyclohexanone oxime electrosynthesis (Figure 5d; Figure S40). The time sequence of representative snapshots directly demonstrates that the ^*^NH_2_OH on the Bi site rapidly nucleophilic attacks ^*^CYC on the Fe site, resulting in the formation of ^*^C_6_H_13_NO_2_, which subsequently undergoes dehydration to yield ^*^CHO. The subsurface Fe atoms serve as the adsorption sites of cyclohexanone, dynamically reconstructing to the surface to facilitate the C─N coupling process. Notably, the coupling reaction between ^*^NH_2_OH and ^*^CYC can occur in the absence of any external field. These results permit the direct visualization of the atomic‐scale synergistic mechanism within Fe_1_Bi SAA. Cyclohexanone and nitrite undergo synergistic adsorption and reduction at the atomic‐scale interfacial Fe and Bi sites, respectively, facilitating the formation of key intermediates ^*^CYC and ^*^NH_2_OH. The synthesis of cyclohexanone oxime is then initiated by the spontaneous C─N bond formation and dehydration. Therefore, the Fe‐Bi atomic interface in Fe_1_Bi SAA facilitates spontaneous, exergonic C─N coupling by optimizing orbital overlap and minimizing kinetic barriers, effectively steering selectivity away from unproductive NH_3_ formation.

## 3 Conclusion

In summary, we have demonstrated a sustainable organonitrogen electrosynthesis route under ambient conditions through the rational design of a Fe_1_Bi SAA electrocatalyst. By atomic‐level engineering of Fe within a Bi host matrix, we constructed a unique architecture that promotes the electrosynthesis of cyclohexanone oxime via direct C─N coupling with exceptional performance on Fe_1_Bi SAA: a FE of 61.3% and a yield rate of 0.27 mmol cm^−2^ h^−1^ with nearly‐100% ketone conversion and oxime selectivity in H‐cell. Moreover, the Fe_1_Bi SAA shows a remarkable FE of 70.9% and a yield rate of 0.94 mmol cm^−2^ h^−1^ in the MEA electrolyzer. The combination of in situ electrochemical spectroscopic investigations and theoretical calculations unveils the atomic‐scale synergistic mechanism: the isolated Fe sites strongly activate cyclohexanone and alter the electronic structure of adjacent Bi sites, which facilitates selective nitrite reduction to the key ^*^NH_2_OH intermediate; the Fe‐Bi atomic interface promotes spontaneous, exergonic C─N coupling toward cyclohexanone oxime electrosynthesis while suppressing over‐reduction to NH_3_ and competing HER. Techno‐economic analysis confirms the industrial viability of this approach, which represents an efficient and scalable route to a critical nylon‐6 precursor. Critically, this work provides new insights for engineering atomically precise interfaces to overcome fundamental limitations in selectivity and activity of multi‐step reactions, opening avenues for sustainable synthesis of high‐value organonitrogen compounds.

## Conflicts of Interest

The authors declare no conflicts of interest.

## Supporting information




**Supporting File**: adma72807‐sup‐0001‐SuppMat.docx.

## Data Availability

The data that support the findings of this study are available from the corresponding author upon reasonable request.
