# Supplementary material for: Inter‐Atomic Synergy on Single‐Atom Alloy Promotes Cyclohexanone Oxime Electrosynthesis
Source: Adv Mater. 2026 Mar 17;38(21):e72807. doi: 10.1002/adma.72807 (PMC13073067; doi:10.1002/adma.72807)
Supplement: Supplementary file 1 — Supporting File: adma72807‐sup‐0001‐SuppMat.docx. [file ADMA-38-e72807-s001.docx]

**Supplementary Information**

**Inter-atomic synergy on single-atom alloy promotes cyclohexanone oxime electrosynthesis**

Panlong Zhai^1^, Chen Wang^2^, Guan Sheng^3^, Chao Ye^3,4^, Jungang Hou^2^, Qinfen Gu^5^, Tao Ling^6^, Ye Zhu^3,*^, Pei Liang^7,*^, Xin Wang^1,*^, Jieqiong Shan^1,8,*^

^1^Department of Chemistry, City University of Hong Kong, Kowloon 999077, Hong Kong SAR, P. R. China

^2^State Key Laboratory of Fine Chemical, School of Chemical Engineering, Dalian University of Technology, Dalian 116024, P. R. China

^3^Department of Applied Physics, Research Institute for Smart Energy, The Hong Kong Polytechnic University, Kowloon 999077, Hong Kong SAR, P. R. China

^4^School of Chemical Engineering, The University of Adelaide, Adelaide, SA 5005, Australia

^5^Australian Synchrotron, ANSTO, Clayton 3168, Australia

^6^School of Materials Science and Engineering, Tianjin University, Tianjin 300072, P. R. China

^7^College of Optical and Electronic Technology, China Jiliang University, Hangzhou 310018, P. R. China

^8^Shenzhen Research Institute, City University of Hong Kong, Shenzhen 518057, P. R. China

**Corresponding authors:** Ye Zhu (yezhu@polyu.edu.hk), Pei Liang (plianghust@cjlu.edu.cn), Xin Wang (wang.xin@cityu.edu.hk), Jieqiong Shan (jieqshan@cityu.edu.hk).

Panlong Zhai, Chen Wang, and Guan Sheng contributed equally to this work.

**Methods section**

**Synthesis of Fe_1_Bi single-atom alloy (Fe_1_Bi SAA)**

Firstly, BiCl_3_ mixture solution was prepared including 10 mmol BiCl_3_, 10 mmol C_6_H_8_O_7_ in 10 mL deionized (DI) water. Then, 1 mL BiCl_3_ mixture solution, 1 mL 0.05 M FeCl_3_·6H_2_O solution, and 1 mL 3 M HCl were added to 17 mL DI water to obtain the precursor solution. The above solution was rapidly added to 10 mL 1 M NaBH_4_ solution in an ice bath and aged for 1 h. After a violent reaction, the obtained precipitate was washed with DI water and ethanol several times, and dried in a vacuum oven overnight.

**Electrocatalysts characterization**

Powder X-ray diffraction patterns (XRD) were characterized on a Bruker D8 Advance X-ray diffractometer with Cu Kα radiation (λ = 1.5406 Å). Scanning electron microscopy (SEM) images were obtained on CIQTEK SEM5000. Transmission electron microscopy (TEM) images and high-angle annular dark-field scanning TEM (HAADF-STEM) images were obtained on JEM-F200 with an accelerating voltage of 200 kV. The AC HAADF-STEM images and EDS characterizations were conducted in a spherical-aberration-corrected TEM (Thermo Scientific Spectra 300 S/TEM) at 300 kV. X-ray photoelectron spectroscope (XPS) data were obtained by Thermo Fisher Scientific ESCALAB250Xi. Ultraviolet-visible (UV-vis) absorption was performed on Shimadzu UV-3600 plus spectrophotometer. In situ Raman experiments were measured by a Raman spectrometer (HORIBA XploRA PLUS), and the laser wavelength is 532 nm. ^1^H NMR spectra were recorded using Bruker Avance 400M NMR spectroscopy. The X-ray absorption spectroscopy (XAS) was conducted in transmission mode at the BL14W1 beamline at the Shanghai Synchrotron Radiation Facility (SSRF, Shanghai, China). Standard data processing was performed by Athena software, including energy calibration and spectral normalization of the raw data.

**Electrochemical measurement**

The electrochemical measurements were conducted on CS310X electrochemical workstation (Wuhan Corrtest Instruments Co., Ltd.) using a typical H-type electrochemical cell with the three-electrode system at room temperature. Typically, the working electrode was prepared as follows: 5 mg as-prepared electrocatalyst and 2 mg Ketjen Black were dispersed in 950 μL ethanol and 50 μL Nafion solution (Dupont, 5 wt%) and sonicated 30 min to form homogeneous ink. Then, 200 μL catalyst ink was drop-casted on carbon fiber paper (CFP, TGP-H-060) with a loading of 1 mg cm^-2^. The potentials were converted to the reversible hydrogen electrode (RHE) scale without iR correction based on the formula: *E*_vs. RHE_ = *E*_vs. Ag/AgCl_ + 0.0592 × pH + 0.197. The Ag/AgCl electrode and Pt net were used as reference electrode and counter electrode. 20 mL of 0.5 M phosphate buffer solution (PBS, pH 5.8) with 0.2 mmol cyclohexanone and 2 mmol NaNO_2_ was used as the catholyte, while 20 mL of 0.5 M PBS (pH 5.8) was used as the anolyte. A proton exchange membrane (Nafion 117) was applied to separate the two compartments of the H-type cell. The linear scanning voltammetry (LSV) test was performed with a scan rate of 10 mV s^-1^. The chronoamperometry was carried out at different applied potentials with stirring, and the reaction time was 2500 s. All the curves were used without iR compensation. The cyclic voltammetry (CV) curves were measured in a non-Faradaic potential window with different scan rates to obtain electrochemical double-layer capacitance (*C*_dl_) and electrochemical active area (ECSA). The electrochemical impedance spectroscopy (EIS) measurements were performed at frequency range from 100 kHz to 0.1 Hz with an amplitude of 5 mV.

**Product quantifications**

The organic products in the electrolyte were identified by gas chromatography-mass spectrometry (GC-MS, Agilent 7890B/5977B) and ^1^H NMR spectroscopy. Cyclohexanone oxime and cyclohexanone in electrolyte were extracted with dichloromethane (DCM) and quantified by GC-MS.

Determination of ammonia (Indophenol blue method): The electrolyte was collected and diluted to the detection range. First, 2 mL of diluted electrolyte was mixed with 2 mL of 1 M NaOH solution with 5 wt% salicylic acid and 5 wt% sodium citrate solution. Subsequently, 1 mL of 0.05 M NaClO and 0.2 mL of 1 wt% sodium nitroferricyanide solution were added. After the mixed solution was left to rest for 2 h under ambient conditions, the absorption spectrum was detected by an ultraviolet-visible spectrophotometer at a wavelength of 655 nm. The calibration curve was made using different concentrations of standard ammonium chloride solution.

Determination of NH_2_OH: The electrolyte was diluted with DI water. 1 mL of diluted electrolyte, 1 mL 0.05 M phosphate buffer solution (pH = 6.8), 0.8 mL DI water, 0.2 mL 12 wt% trichloroacetic acid aqueous solution, 1 mL 0.01 g L^-1^ 8-quinolinol ethanol solution, and 1 mL 1 M Na_2_CO_3_ aqueous solution were added sequentially. The final mixture was heated at 100 ℃ for 1 min. After cooling to room temperature, the absorption spectrum was detected by an ultraviolet-visible spectrophotometer at a wavelength of 705 nm.

FE of products was calculated as follows:

$$\text{FE= }\frac{\text{mol of product × n ×F}}{\text{Q}}\text{ ×100\%}$$

Where n is the electron transfer number, F is the Faraday constant (96485 C mol^-1^), and Q is the total charge passed through the electrode.

The yield was calculated as follows:

$$\text{Yield= }\frac{\text{mol of the formed product }}{\text{mol of the initial of substrate}}\text{ ×100\%}$$

The yield rate was calculated as follows:

$$\text{Yield rate = }\frac{\text{mol of the formed product }}{\text{mol of the initial of substrate ×t ×S}}\text{ ×100\%}$$

Where t is the reaction time and S is the geometric area of the electrode.

**MEA electrolyzer**

In the MEA electrolyzer with the electrode area of 1 cm^2^ (1 cm × 1 cm), NiFe LDH/nickel foam was used as anode and Fe_1_Bi SAA sprayed on carbon fiber paper was used as cathode, which was separated by a proton exchange membrane (Nafion 117). The gasket was placed between the plate and membrane. The anolyte was 1 M KOH and the catholyte was 0.5 M PBS (pH = 5.8) containing 0.5  M NaNO_2_ and 0.05 M cyclohexanone.

**In situ ATR-SEIRAS measurement**

The in situ ATR-SEIRAS were measured by BRUKER INVENIO spectrometer with an MCT detector and VeeMAX III ATR accessory. The Au films were chemically deposited on the silicon crystal. The sample was drop-casted on the Au-deposited Si crystal for the working electrode of the custom-made spectroelectrochemical cell fixed on the ATR accessory. The Ag/AgCl and Pt wire were used as reference and counter electrode, respectively. The electrolyte was 0.5 M PBS (pH 5.8) with 0.1 M NaNO_2_ and 0.01 M cyclohexanone. The background was measured at open circuit potential. Afterward, the spectra at different potentials were collected with 4 cm^-1^ resolution and scan number of 64.

**Online DEMS measurements**

The DEMS experiments were conducted using QAS 100 mass spectrometer (Shanghai Linglu Instruments). The DEMS experiments were measured with a homemade electrochemical cell, in which Pt wire and Ag/AgCl electrode serve as the counter electrode and reference electrode, respectively. The working electrode was prepared by drop-casting the catalyst ink on a gold-sputtered PTFE membrane with a mass loading of 0.5 mg cm^-2^. The mass signal was detected when the electrodes were subjected to potential cycling. The products generated during the reaction were detected by the real-time mass spectrometry.

**Techno-economic analysis**

To determine the economic potential of renewable electricity powered production of cyclohexanone oxime through electrocatalytic reductive C-N coupling, we conducted a techno-economic analysis (TEA) based on models from previous studies.^[1,2]^

Below is the list of specific assumptions made for the calculations.

1. The production capacity of the plant is 1 tonne of cyclohexanone oxime per day.

2. The lifetime of the electrolyzer is to be 20 years with no salvage value at the end of the plants lifetime.

3. The Faradaic efficiency to cyclohexanone oxime is 64%, the cell operating voltage is 2.4 V, and the total operating current density is 100 mA cm^-2^.

4. Separation equipment costs will be assumed to be 10% of the electrolyzer costs. The catalyst and membrane cost is 5% of the electrolyzer cost.

5. The price of electricity is $ 0.05 /kWh.

6. The cost of Balance of Plant was set as 35% of the cost of the electrolyzer.

7. The separation cost is assumed to be 20% of the electricity cost.

8. The maintenance cost was set at 2.5% of the electrolyzer cost.

9. The operation costs are assumed to be 10% of the electricity cost.

10. The price of cyclohexanone and nitrite is assumed to be $1161 per tonne and $593 per tonne, respectively.

The total current needed is:

$$\text{Total current = 1000 }\frac{\text{kg}}{\text{day}}\text{ × }\frac{\text{day}}{\text{86400 s}}\text{ × }\frac{\text{1000 g}}{\text{kg}}\text{ × }\frac{\text{mol}}{\text{113.16 g}}\text{×4}\text{e}^{\text{-}}\text{ × }\frac{\text{96485 C}}{\text{mol}}\text{ × }\frac{\text{1}}{\text{64\%}}\text{= 61678.4 A}$$

The total electrolyzer area needed is:

$$\text{Total }\text{electrolyzer}\text{ area = }\frac{\text{61678.4 A}}{\text{0.1 A }\text{cm}^{\text{-2}}}\text{ × }\frac{\text{m}^{\text{2}}}{\text{10}^{\text{4}}\text{ }\text{cm}^{\text{2}}}\text{=61.7 }\text{m}^{\text{2}}$$

The power needed is calculated by P = UI

$$\text{Total power needed = 2.4 V × 61678.4 A × }\frac{\text{kW}}{\text{10}^{\text{3}}\text{ W}}\text{=148.0 kW}$$

Based on the E4tech/Element Energy report and DOE H2A analysis for central grid electrolysis, the electrolyzer cost for the stack component is $550/kW with reference electrolyzer current of 0.4 A/cm^2^ and 1.75 V.^[3]^ An installation factor is 1.12. Thus, the total cost of the electrolyzer is:

$$\text{Total }\text{Electrolyzer}\text{ cost= }\frac{\text{550 }\text{kW}^{\text{-1}}}{\text{1000}\frac{\text{W}}{\text{kW}}}\text{ ×0.4 A }\text{cm}^{\text{-2}}\text{ ×1.75 V × }\frac{\text{61.7}\text{m}^{\text{2}}}{\text{0.0001}\frac{\text{m}^{\text{2}}}{\text{cm}^{\text{2}}}}\text{ ×1.12= \$ 266050.4}$$

Considering the capital recovery factor (CRF) based on a 5% discount rate (denote i in the equation below), with a lifetime of 20 years:

$$\text{CRF= }\frac{\text{i(1+i)}^{\text{year}}}{\text{i(1+i)}^{\text{year}}\text{ - 1}}\text{=0.08}$$

The electrolyzer cost per ton of cyclohexanone oxime is:

$$\text{Electrolyzer}\text{ cost= \$ 266050.4 × }\frac{\text{0.08}}{\text{350 day}}\text{ ×}\frac{\text{1}}{1 \frac{\text{tonne}}{\text{day}}}\text{= \$ 60.8 }\text{tonne}^{\text{-1}}$$

Separation equipment cost:

$$\text{Separation equipment cost= \$ 60.8 }\text{tonne}^{\text{-1}}\text{ ×0.1= \$ 6.1 }\text{tonne}^{\text{-1}}\text{ }$$

Catalyst and membrane cost:

$$\text{Catalyst and membrane cost= \$ 60.8 }\text{tonne}^{\text{-1}}\text{ ×0.05= \$ 3.0 }\text{tonne}^{\text{-1}}$$

Balance of Plant:

From the H2A, the balance of plant cost is 35% of the electrolyzer, while the stack is 65%:

$$\text{Balance of Plant = \$ 60.8 }\text{tonne}^{\text{-1}}\text{ × }\frac{\text{0.35}}{\text{0.65}}\text{ = \$ 32.7 }\text{tonne}^{\text{-1}}$$

Capital cost:

$$\text{Capital cost= \$ (60.8 + 6.1 + 3.0 + 32.7) }\text{tonne}^{\text{-1}}\text{ = \$ 102.6 }\text{tonne}^{\text{-1}}$$

Electricity cost:

$$\text{Electricity cost= 148.0 kW × 24}\frac{\text{h}}{\text{day}}\text{ × }\frac{\text{\$ 0.05}}{\text{kWh}}\text{ × }\frac{\text{1}}{1 \frac{\text{tonne}}{\text{day}}}\text{=\$ 177.6 }\text{tonne}^{\text{-1}}$$

Separation cost:

$$\text{Separation equipment cost= \$ 177.6 }\text{tonne}^{\text{-1}}\text{ ×0.2= \$ 35.5 }\text{tonne}^{\text{-1}}\text{ }$$

Maintenance:

$$\text{Maintenance = \$ 60.8 }\text{tonne}^{\text{-1}}\text{ ×0.025= \$ 1.5 }\text{tonne}^{\text{-1}}$$

Operation cost:

$$\text{Operation cost = \$ 177.6 }\text{tonne}^{\text{-1}}\text{ ×0.1= \$ 17.8 }\text{tonne}^{\text{-1}}$$

Input materials cost:

$\text{Input material cost = \$ 1161 × 0.867 + \$ 593 × 0.610 = \$ 1368.}$3$\text{ }\text{tonne}^{\text{-1}}$

The total cost:

The plant-gate levelized cost to produce 1 tonne of cyclohexanone oxime can be calculated by summing up all costs.

$$\text{Total cost = 102.6 + 177.6 + 35.5 + 1.5 + 17.8 + 1368.3 = \$ 1703.3 }\text{tonne}^{\text{-1}}$$

**Theoretical Calculation**

First-principles calculations were performed based on density functional theory (DFT) using PWmat code.^[4]^ The electronic ion interaction was described in the projector augmented wave (PAW) method. The Perdew-Burke-Ernzerhof generalized gradient approximation (PBE-GGA) was utilized as the exchange correlation functional.^[5,6]^ NCPP-SG15-PBE pseudopotential is used for PBE calculation. A plane wave basis set with a cutoff of 60 Ryd is used. The convergence criteria for the energy and force were set to 10^-5^ eV and 10^-3^ eV Å^-1^, respectively. The k-point meshing for the Brillouin zone was set up using a 4×4×1 gamma-centered grid generated by the Monkhorst-Pack Scheme.

For the ab initio molecular dynamics (AIMD) simulations, the Vienna Ab initio Simulation Package (VASP version 6.3)^[7]^ is used for the AIMD. A surface temperature of 300 K is used, where the atoms in the top four layers are allowed to move in all three directions, and the ideal lattice constant is expanded by a factor of 1.0001 in order to reflect the expansion of the bulk due to the surface temperature. The surface is modeled using an 8 layer (4*4) supercell. For each AIMD data point, 1000 trajectories were run with a time step of 0.5 fs. Moreover, the van der Waals (vdW) correlation functional developed by Dion and coworkers (vdW-DF1) was employed for the correlation component.^[8]^


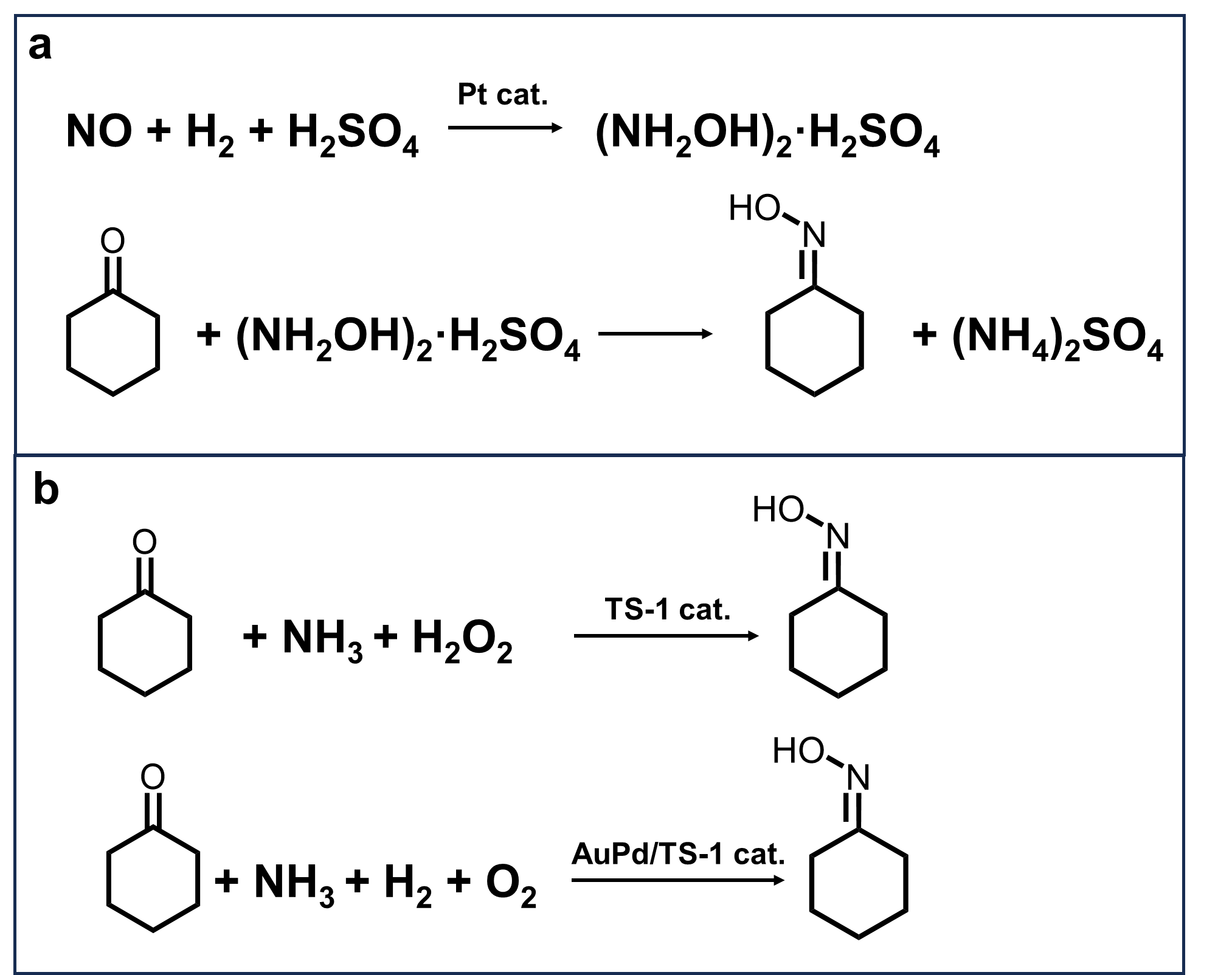


**Figure S1**. (a) Cyclohexanone-hydroxylamine method by reacting with hydroxylamine sulfate. (b) Cyclohexanone ammoximation method by exogenous H_2_O_2_ or in situ-generated H_2_O_2_ from H_2_ and O_2_.


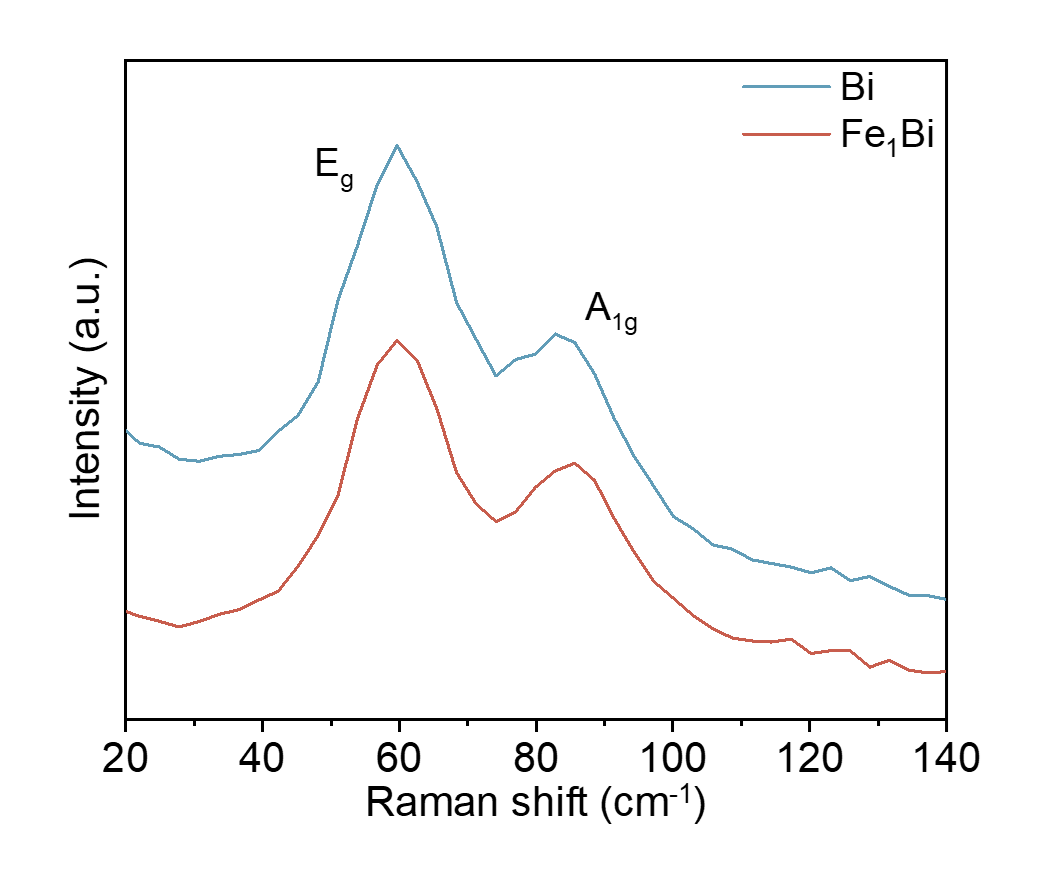


**Figure S2**. Raman spectra of pristine Bi and Fe_1_Bi SAA.

.
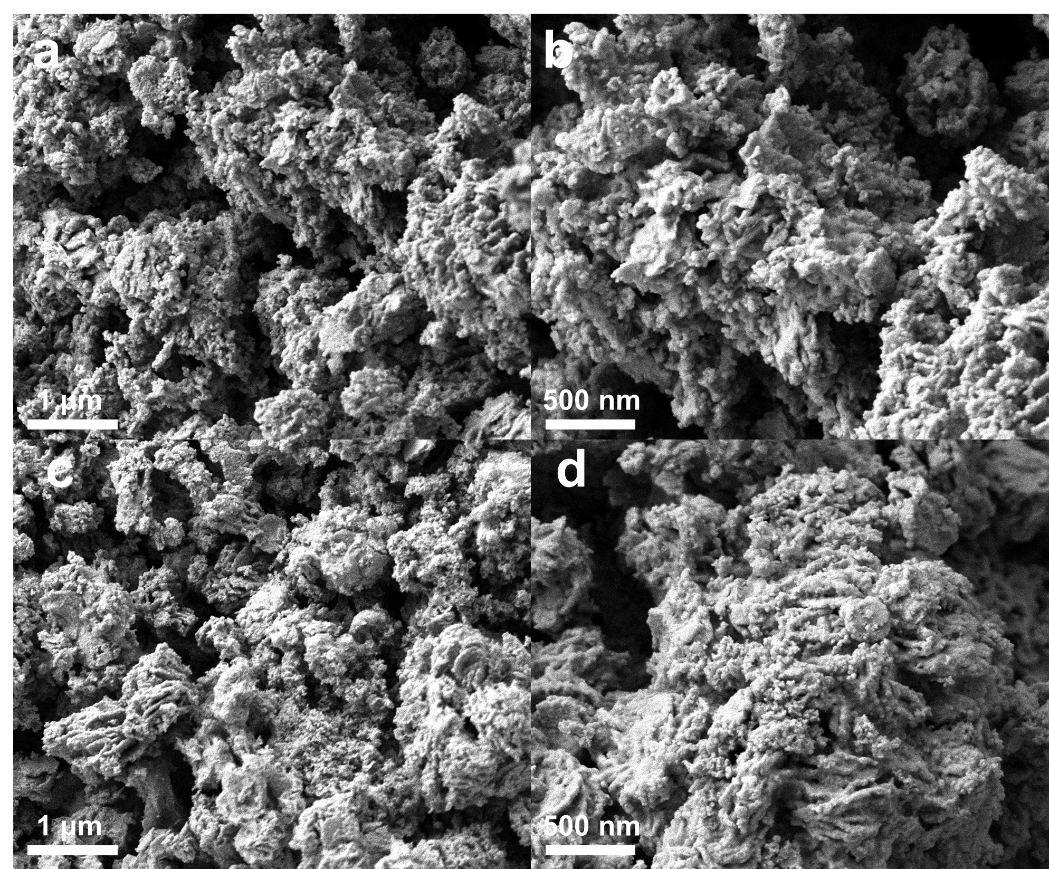


**Figure S3**. SEM images of (a,b) pristine Bi and (c,d) Fe_1_Bi SAA at different magnifications.


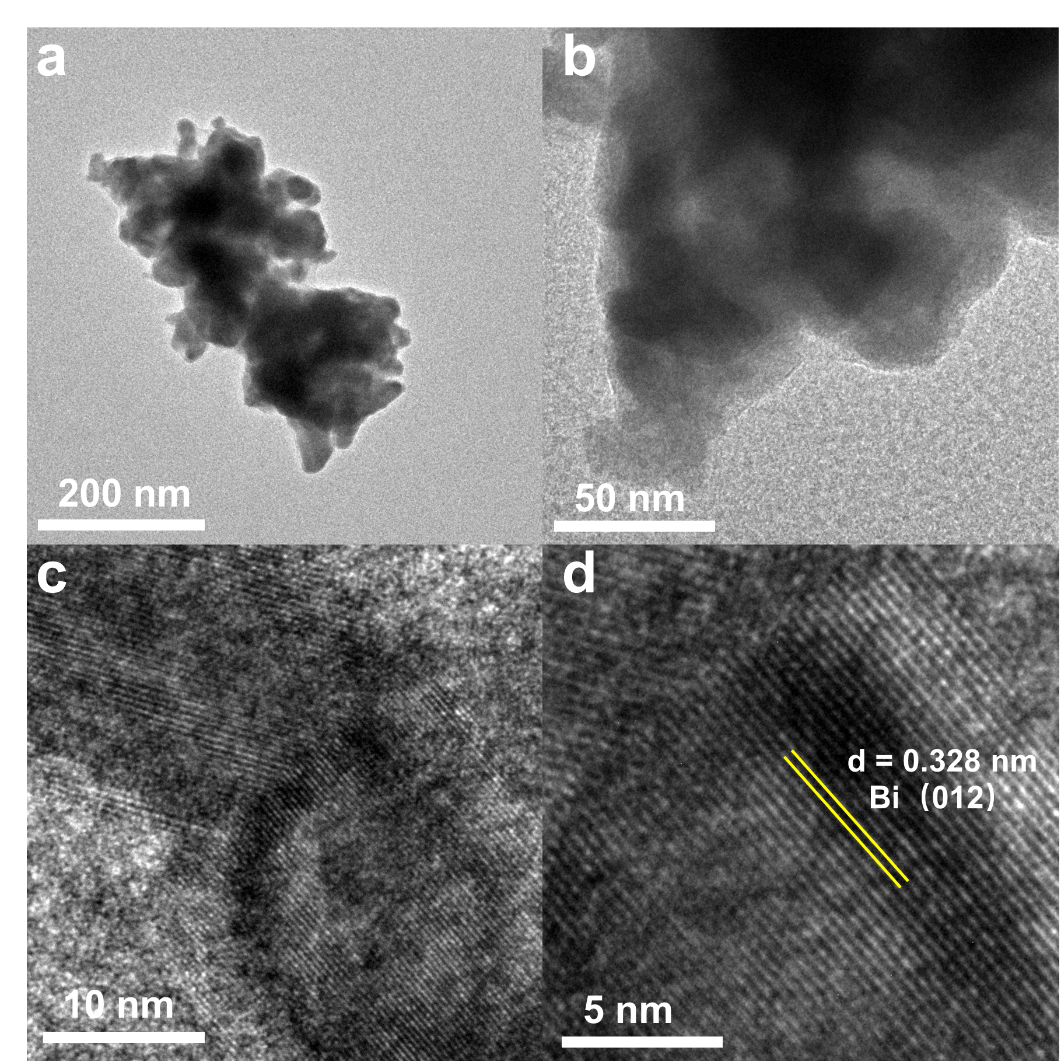


**Figure S4.** (a,b) TEM images and (c,d) HR-TEM images of pristine Bi at different magnifications.


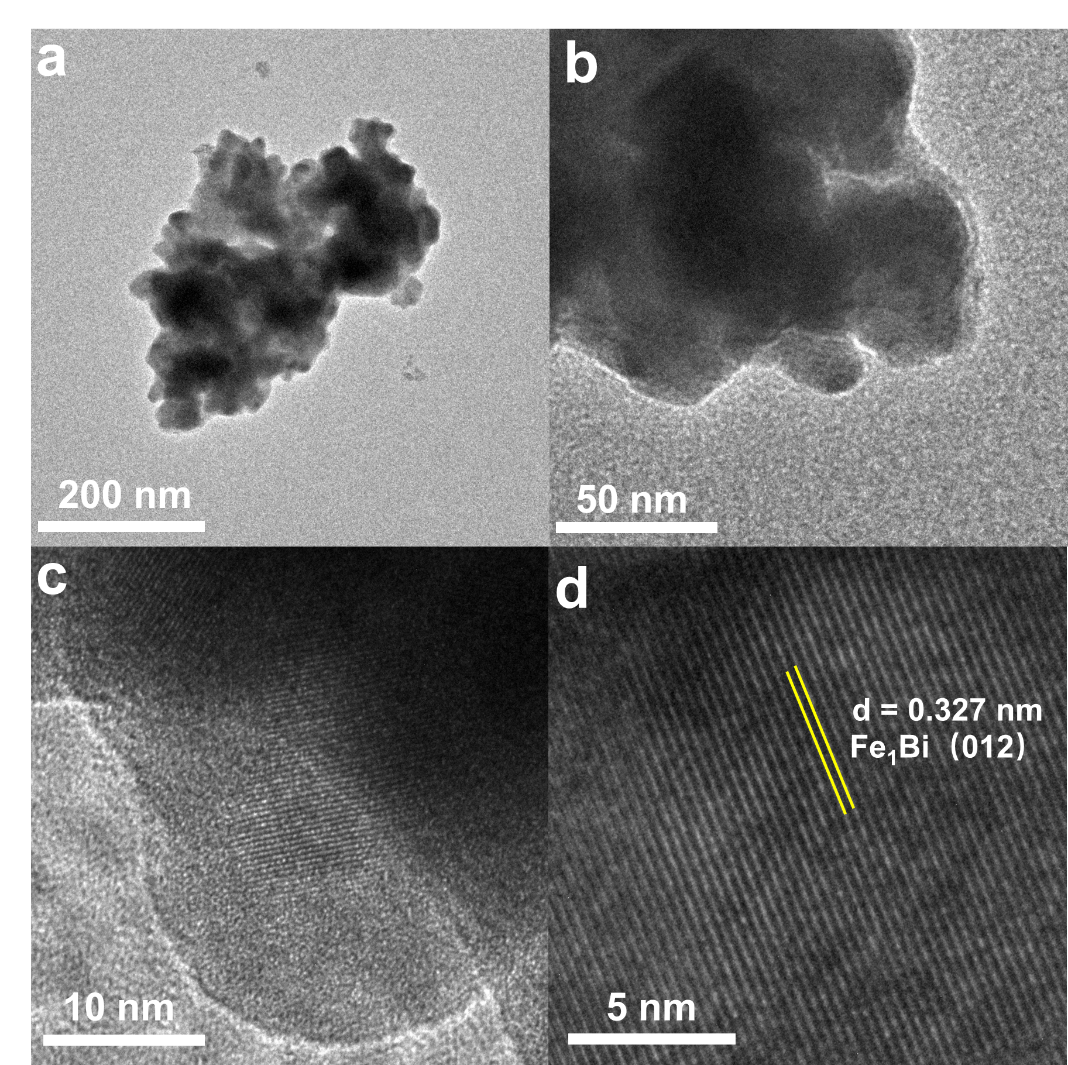


**Figure S5**. (a,b) TEM images and (c,d) HR-TEM images of Fe_1_Bi SAA at different magnifications.


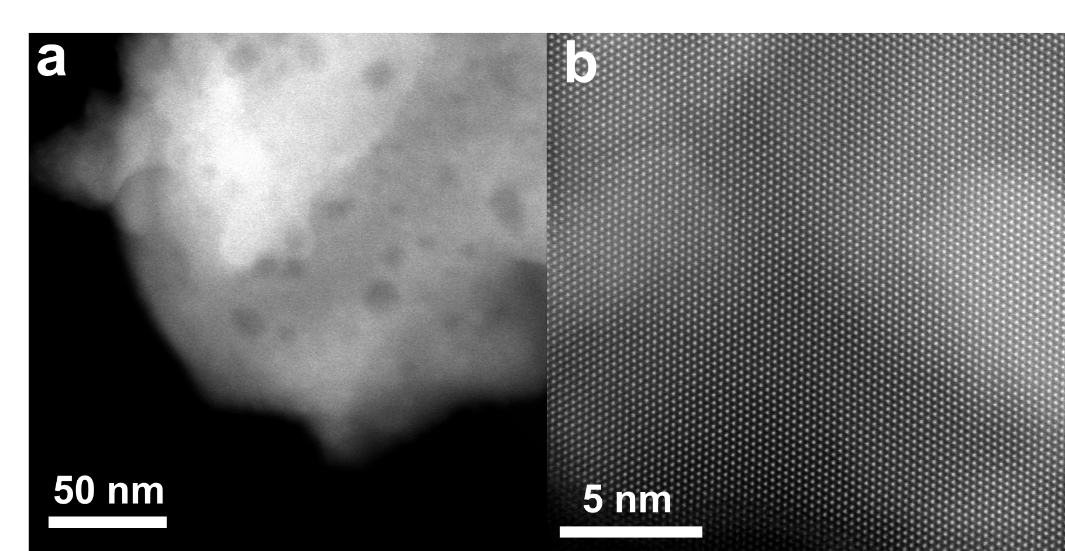


**Figure S6**. (a,b) AC HAADF-STEM images of Fe_1_Bi SAA at different magnifications.


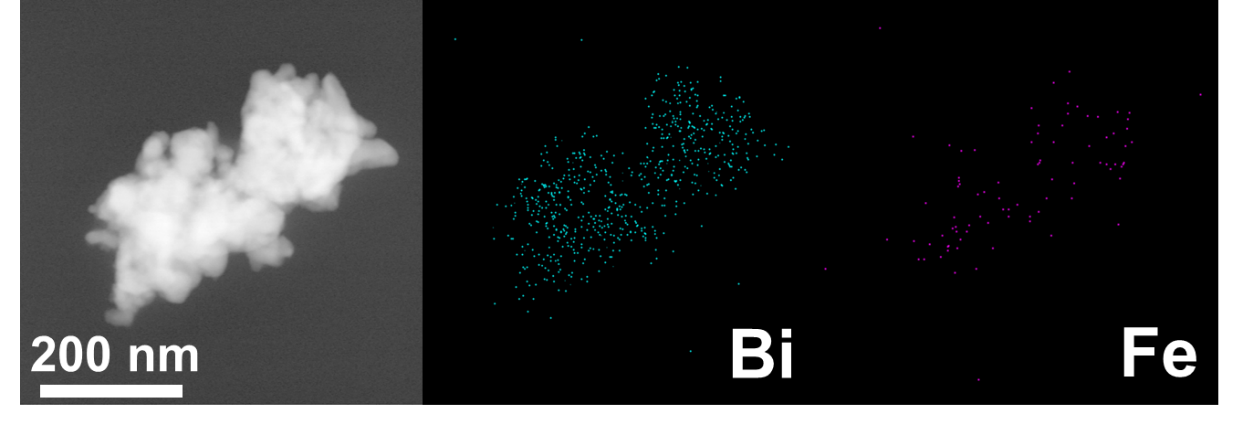


**Figure S7**. Elemental mapping of Fe_1_Bi SAA.


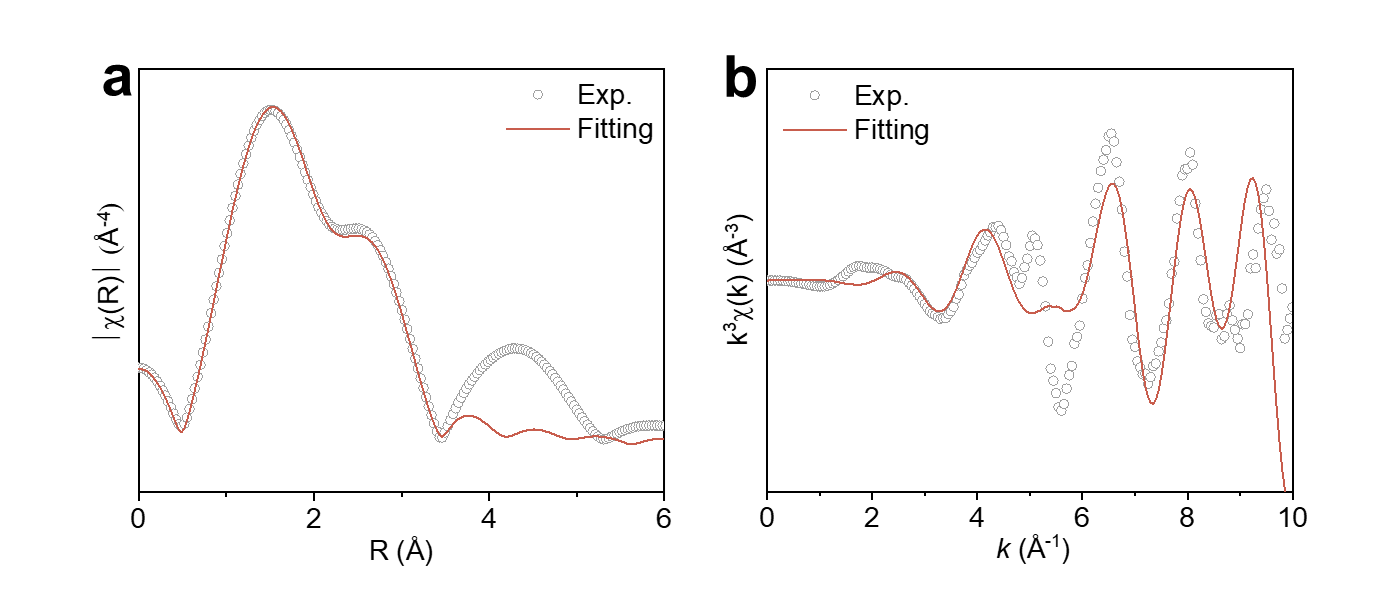


**Figure S8**. (a) FT-EXAFS and corresponding fitting curves of Fe_1_Bi SAA. (b) EXAFS and corresponding fitting curves of Fe_1_Bi SAA.


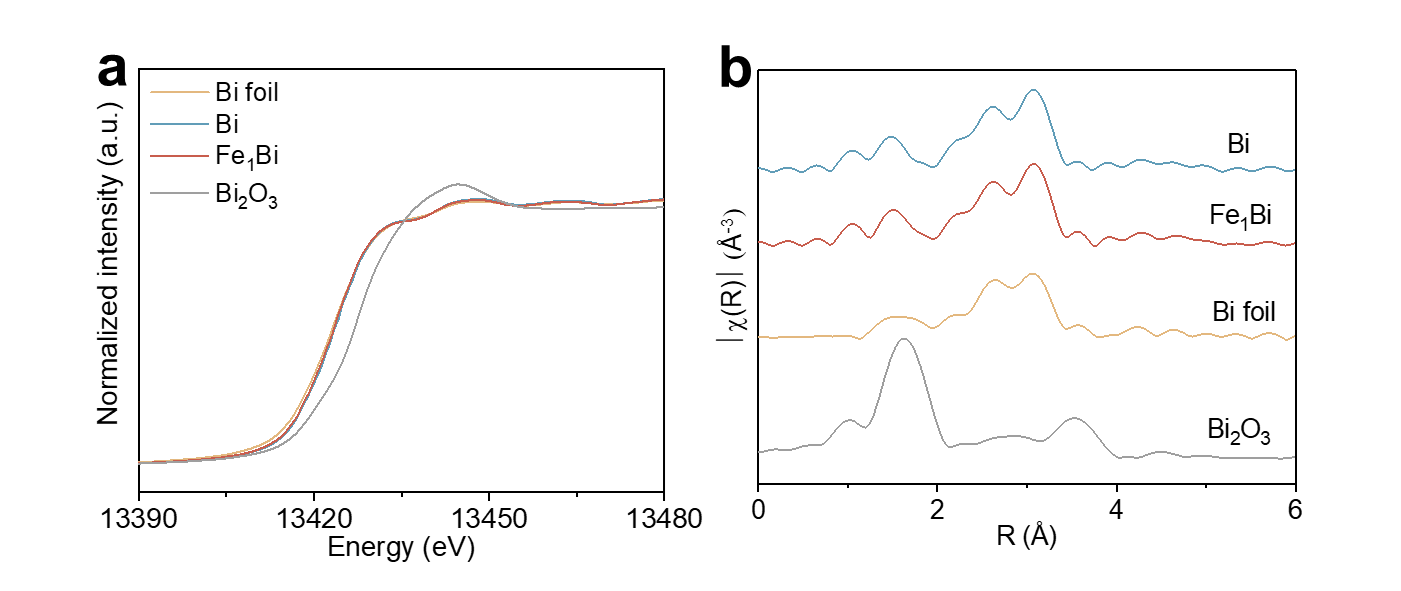


**Figure S9**. (a) Bi L_Ⅲ_-edge XANES spectra and (b) Fourier-transformed EXAFS spectra of pristine Bi, Fe_1_Bi SAA and the reference.

The FT-EXAFS spectra of Fe_1_Bi SAA show that the characteristic peaks at 2.6 and 3.1 Å are assigned to the intralayer and interlayer of Bi-Bi bonds, respectively, while the peak at 1.5 Å is attributed to the Bi-O bond due to the surface oxidation by exposure to air.


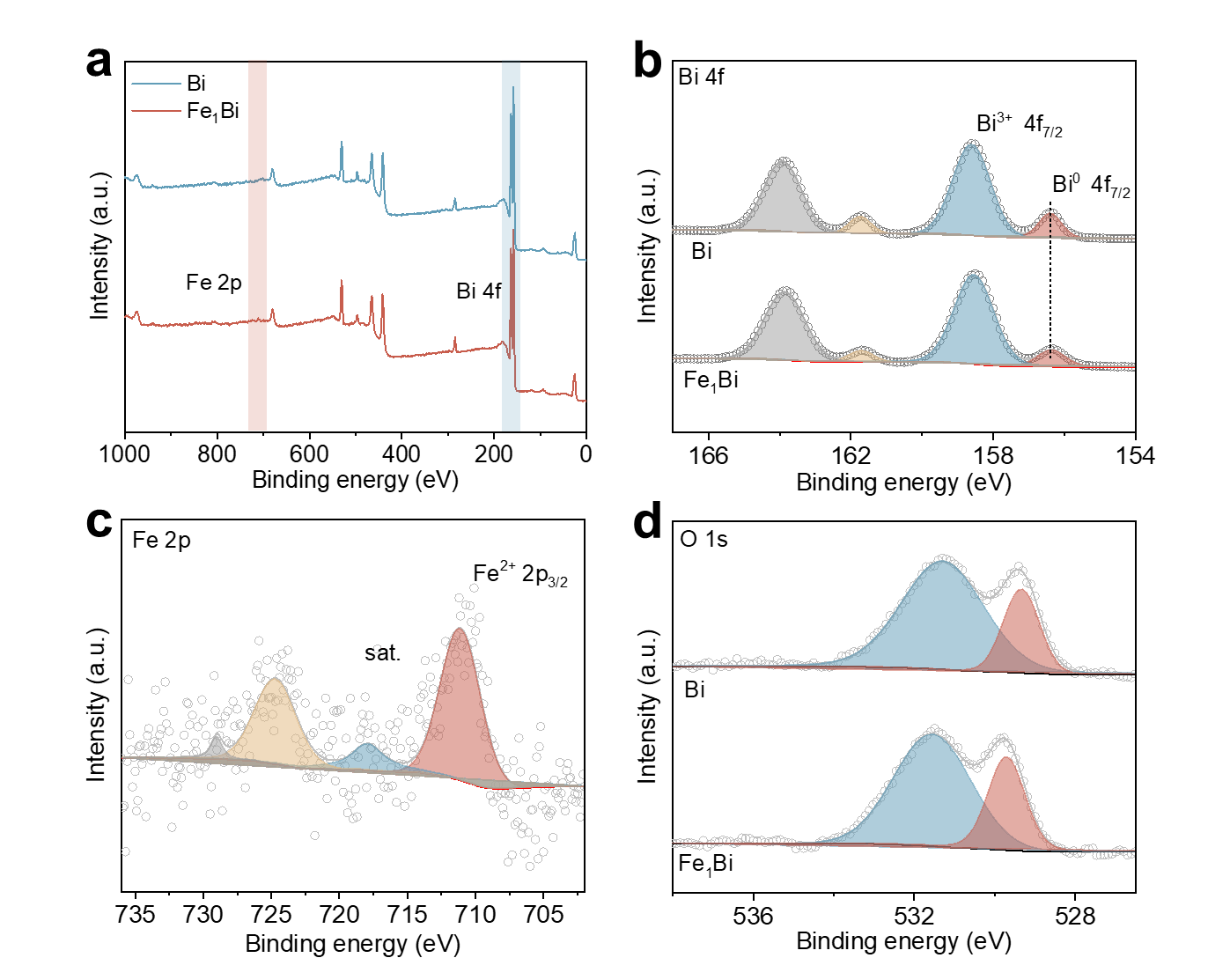


**Figure S10.** (a) XPS survey spectra for pristine Bi and Fe_1_Bi SAA. High-resolution (b) Bi 4f, (c) Fe 2p and (d) O 1s XPS spectra for pristine Bi and Fe_1_Bi SAA.

The high-resolution Bi 4f spectra of Fe_1_Bi SAA show peaks at 156.3 and 158.6 eV, which are assigned to metallic Bi and Bi^3+^, respectively. Compared to Bi, the binding energy of pristine Bi in Fe_1_Bi SAA is negatively shifted by 0.1 eV, indicating the strong electronic interaction and charge transfer between Fe and Bi (Figure S10b). The high-resolution Fe 2p spectra exhibit binding energies located at 711.1 and 724.8 eV, which are assigned to the Fe 2p_3/2_ and Fe 2p_1/2_ of Fe^2+^ species (Figure S10c). The high-resolution O 1s XPS spectra of pristine Bi and Fe_1_Bi SAA display similar binding energies at 529.4 and 531.3 eV, which are assigned to adsorbed hydroxide and adsorbed water, respectively (Figure S10d).

**Figure S11**. ^1^H NMR spectra of cyclohexanone oxime in 0.5 M PBS and 0.5 M H_2_SO_4_.

The ^1^H NMR spectra show that cyclohexanone oxime hydrolyzes rapidly in acidic solution within ~10 min, leading to non-electrochemical loss of the nitrogen feedstock.

**Figure S12**. LSV curves of Bi in different electrolytes.


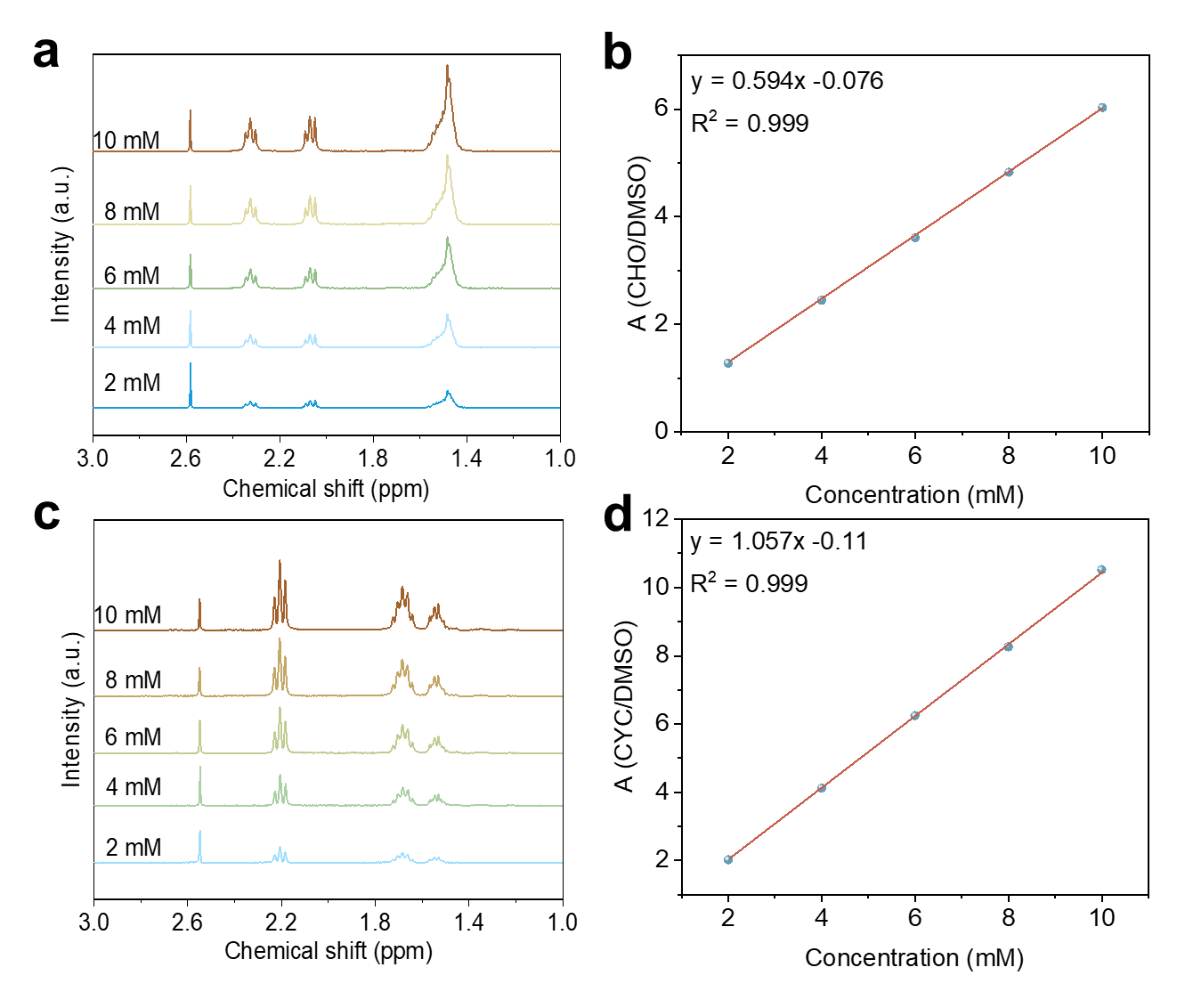


**Figure S13**. The ^1^H NMR spectra of (a) cyclohexanone oxime and (c) cyclohexanone with different concentrations. The standard curve of integral area against (b) cyclohexanone oxime and (d) cyclohexanone.


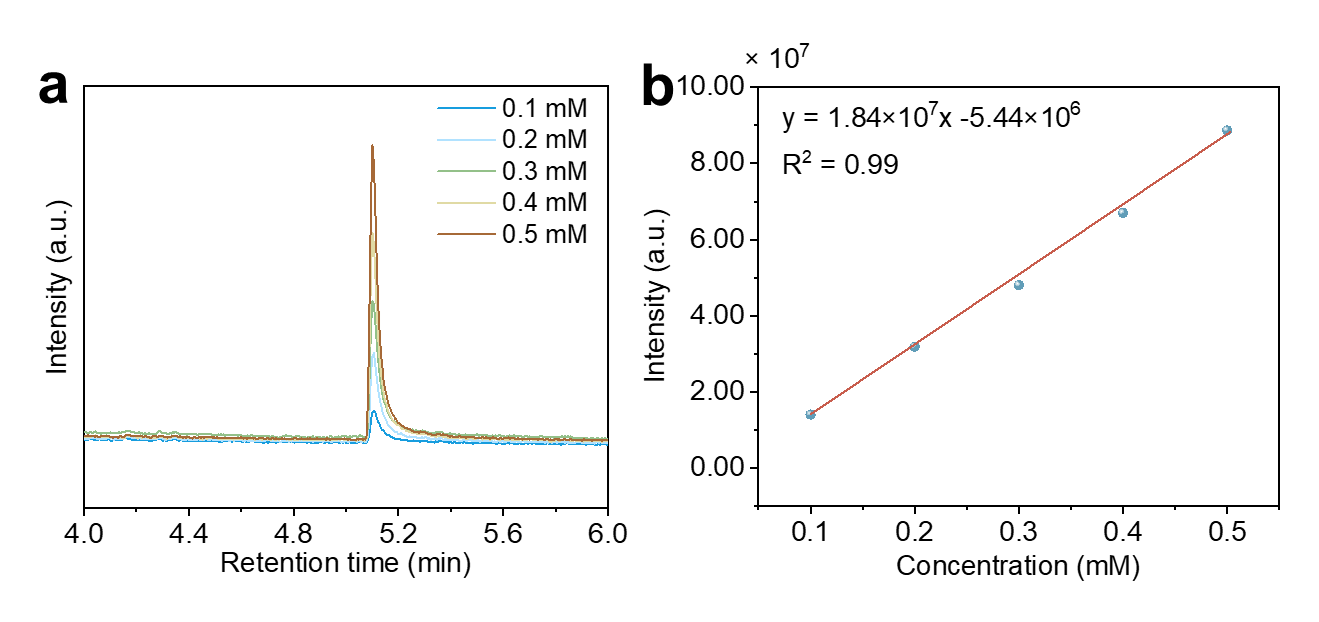


**Figure S14**. (a) The GC-MS spectra of cyclohexanone oxime. (b) The standard curve of integral area against cyclohexanone oxime.


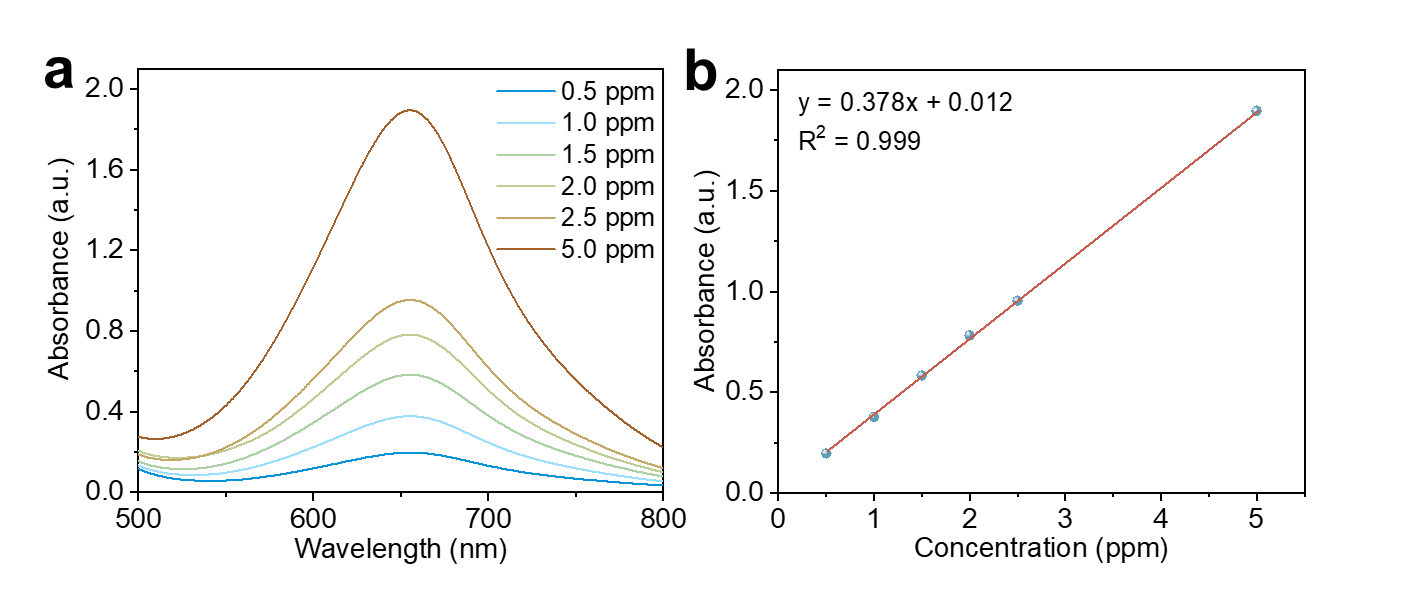


**Figure S15**. (a) UV-Vis curves of NH_4_^+^ as the standard solution and (b) linear fitting results of the calibration curves at 655 nm.


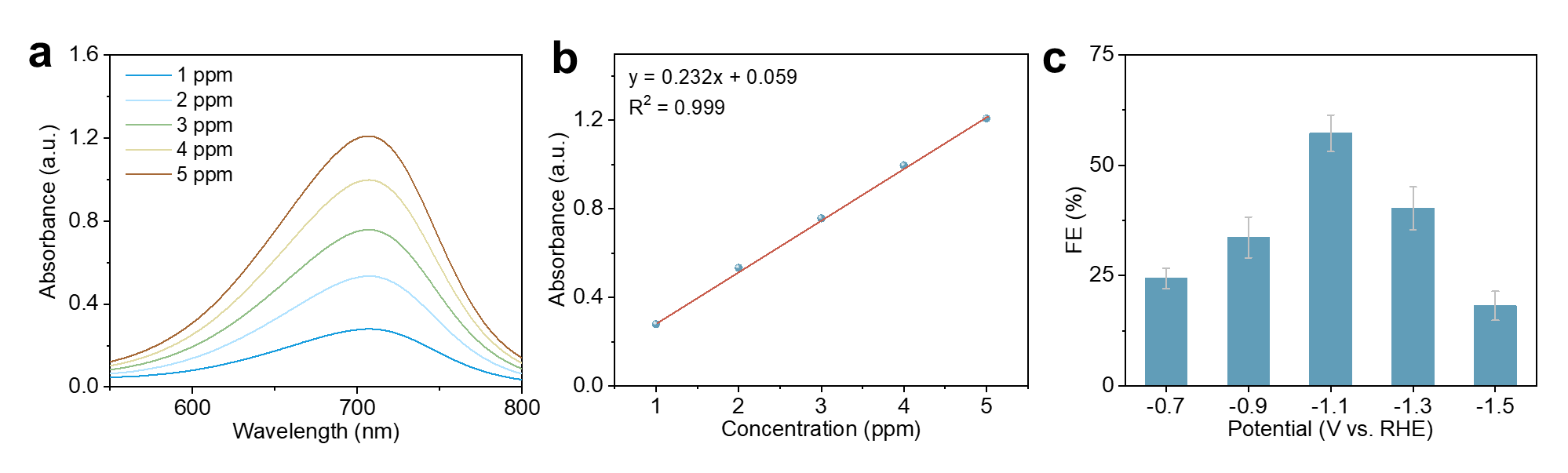


**Figure S16**. (a) UV-Vis curves of NH_2_OH as the standard solution and (b) linear fitting results of the calibration curves at 705 nm. (c) FE of NH_2_OH for Fe_1_Bi SAA without cyclohexanone.

Control experiments for nitrite reduction were conducted in the absence of cyclohexanone. The FE for NH_2_OH generation on Fe_1_Bi SAA shows a volcano-type trend with applied potential, reaching up to 57.2% at −1.1 V vs. RHE, confirming that free NH_2_OH can be synthesized and accumulate in the electrolyte. In contrast, after the addition of cyclohexanone, no NH_2_OH is detected in the post-electrosynthesis electrolytes, indicating its rapid consumption by cyclohexanone to form oxime product.


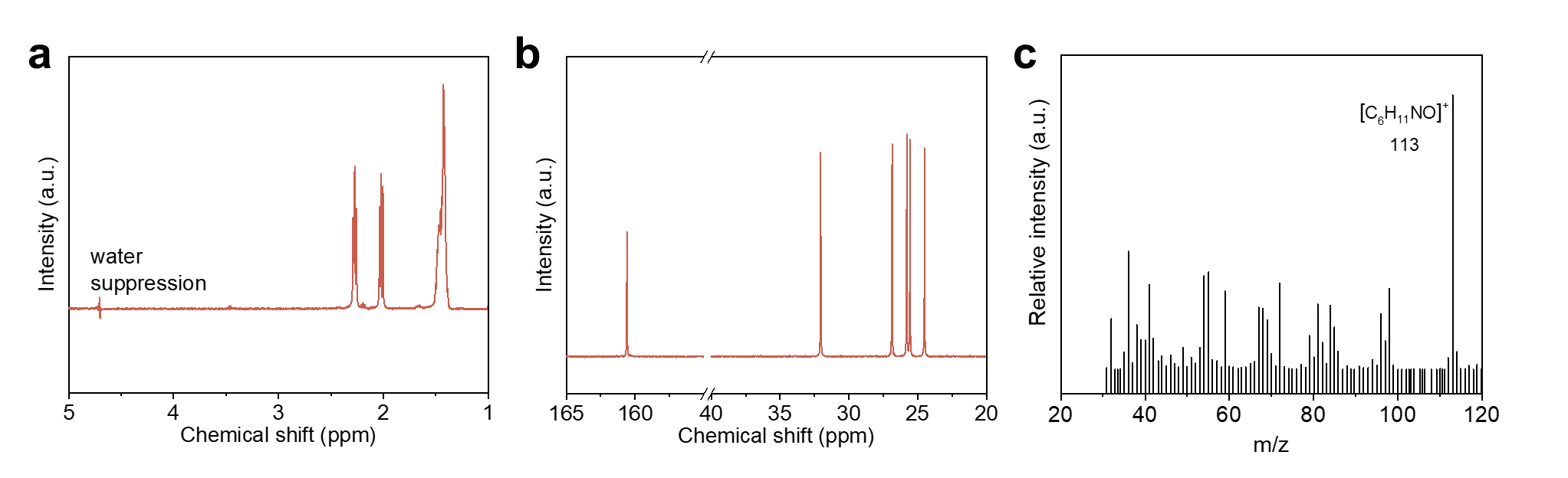


**Figure S17**. (a) ^1^H NMR and (b) ^13^C NMR spectra of cyclohexanone oxime product. (c) GC-MS of synthesized cyclohexanone oxime.

The ^1^H NMR and ^13^C NMR spectra identified that cyclohexanone oxime is the exclusive organic product.

**Figure S18**. Yield of cyclohexanone oxime for Fe_1_Bi SAA and pristine Bi.


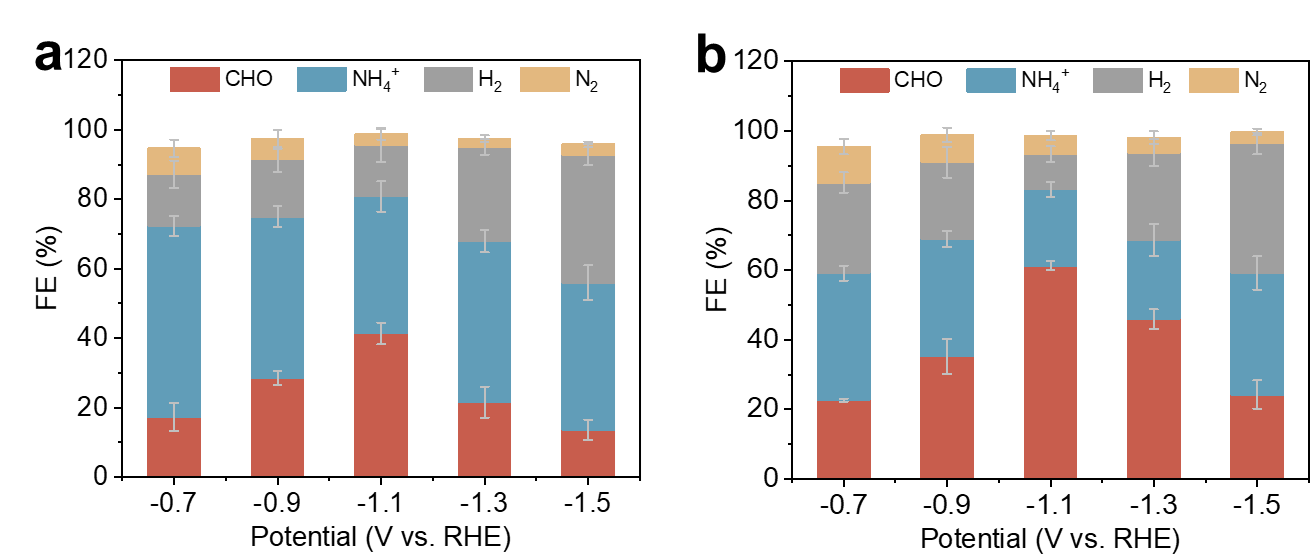


**Figure S19**. Potential dependent FE of products over (a) pristine Bi and (b) Fe_1_Bi SAA.


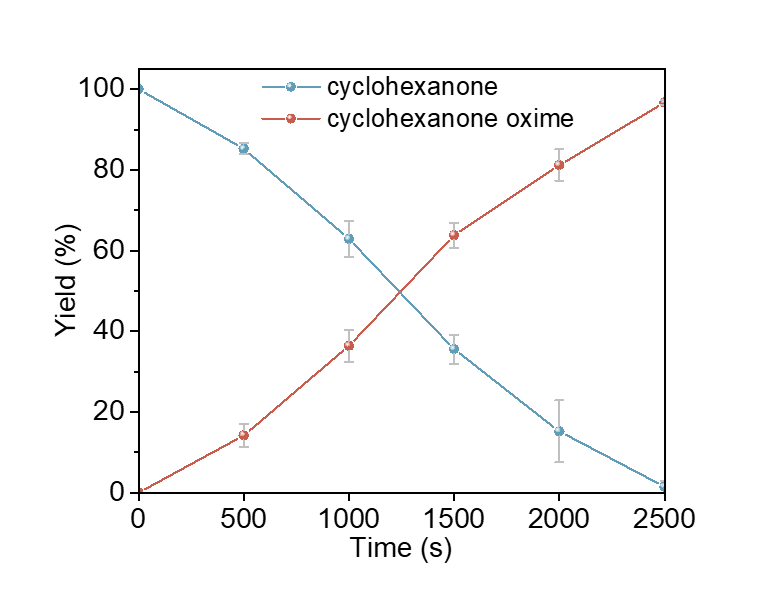


**Figure S20.** Time-dependent conversion of cyclohexanone and yield of cyclohexanone oxime.


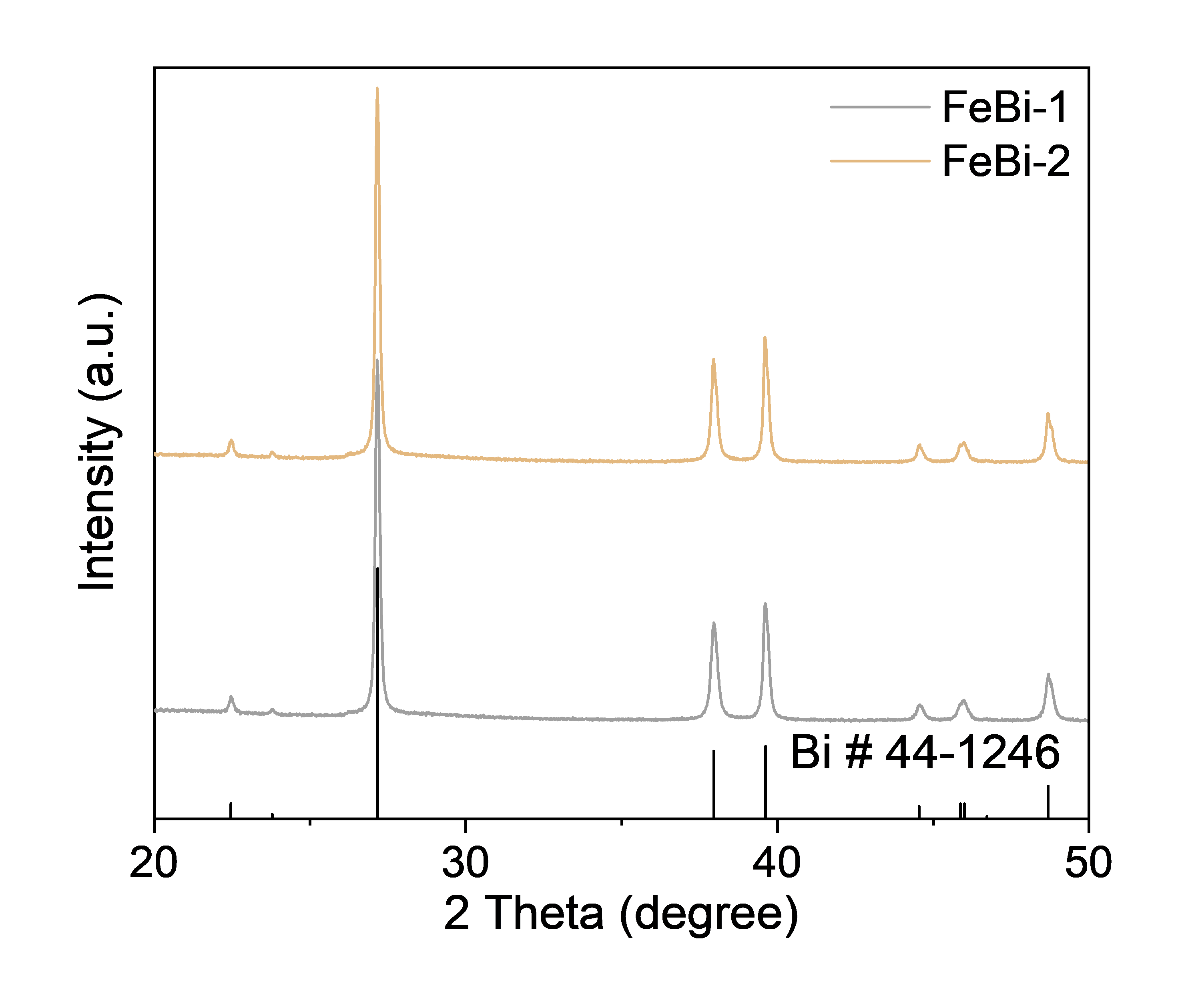


**Figure S21**. XRD patterns of FeBi-1 and FeBi-2.


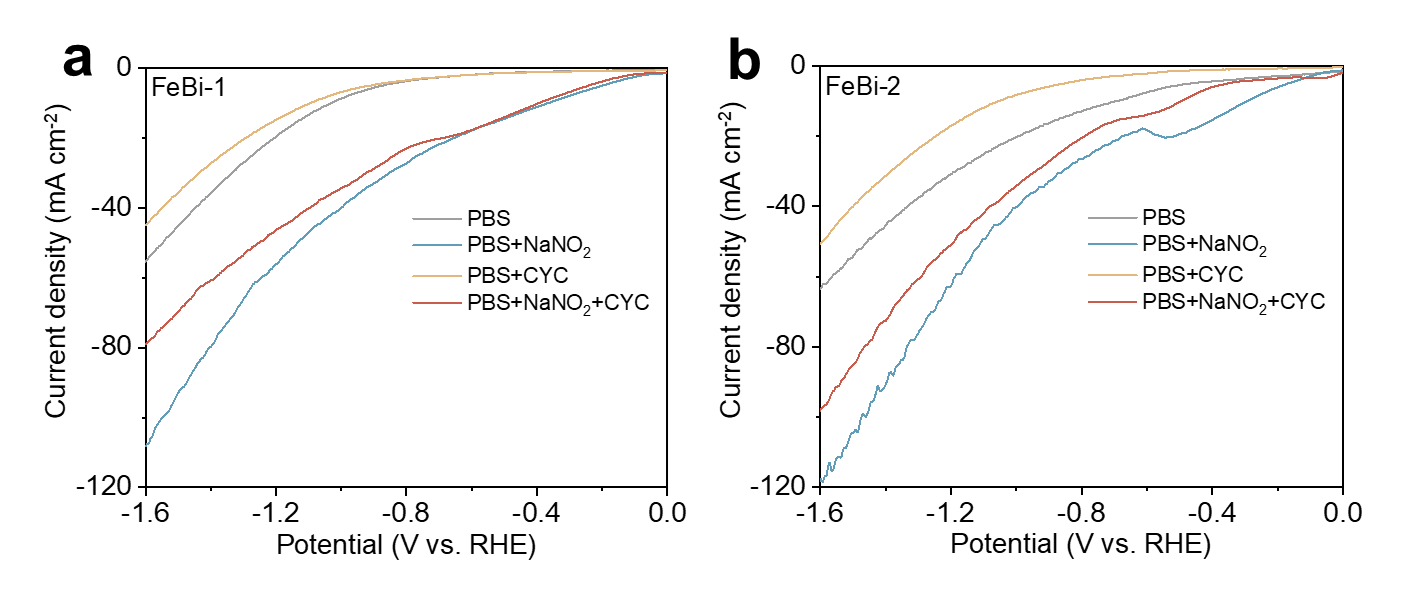


**Figure S22**. LSV curves of (a) FeBi-1 and (b) FeBi-2 in different electrolytes.


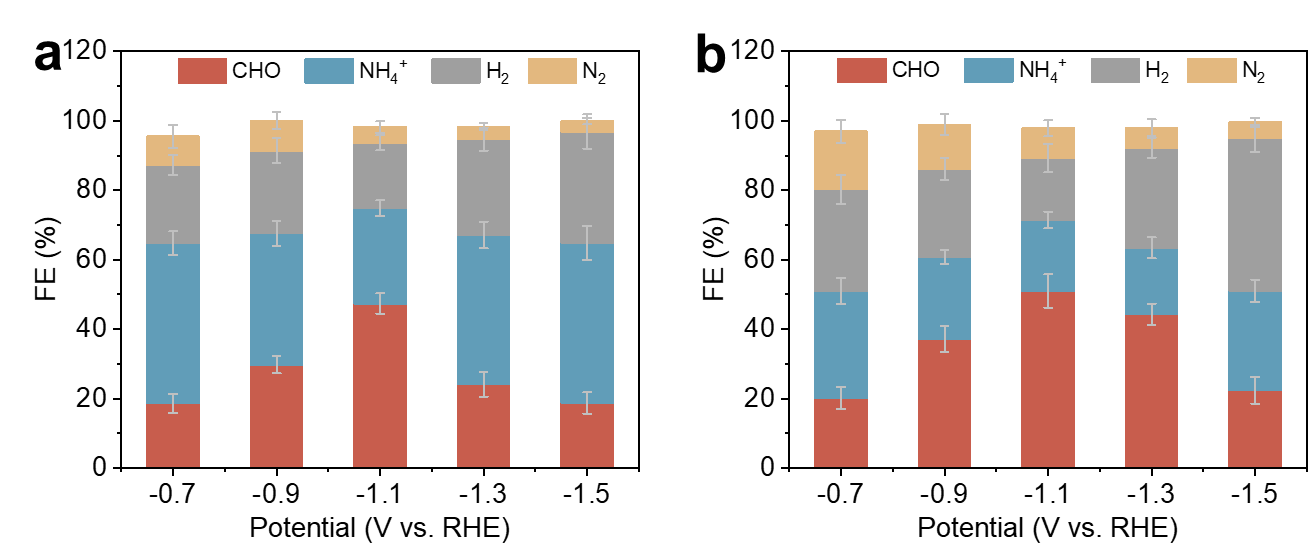


**Figure S23**. Potential dependent FE of products over (a) FeBi-1 and (b) FeBi-2.


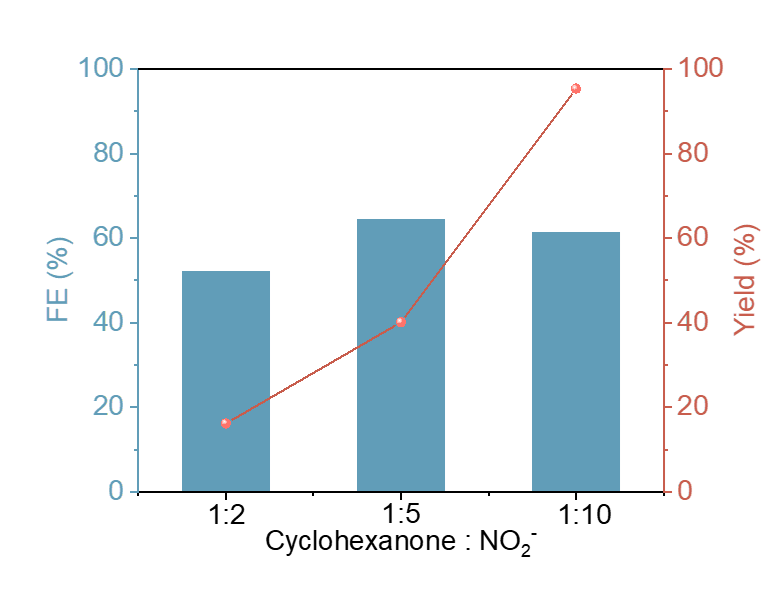


**Figure S24**. Cyclohexanone oxime yield and FE under the conditions of different molar ratios of cyclohexanone and nitrite.


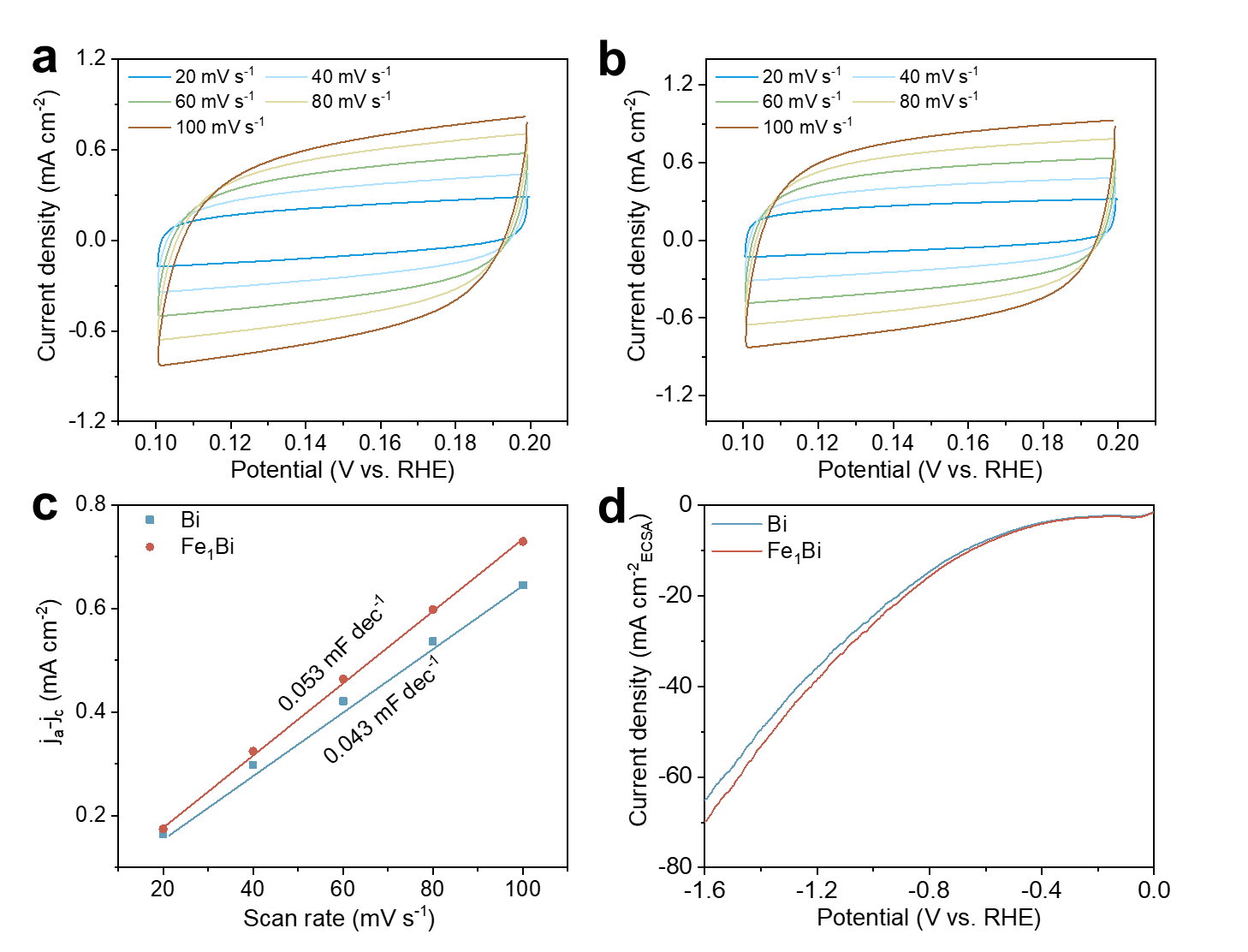


**Figure S25**. The cyclic voltammetry curves at various scan rates of (a) pristine Bi and (b) Fe_1_Bi SAA. (c) Double-layer capacitance for pristine Bi and Fe_1_Bi SAA. (d) Polarization curves with current density normalized to ECSA.

**Figure S26**. EIS of pristine Bi and Fe_1_Bi SAA.


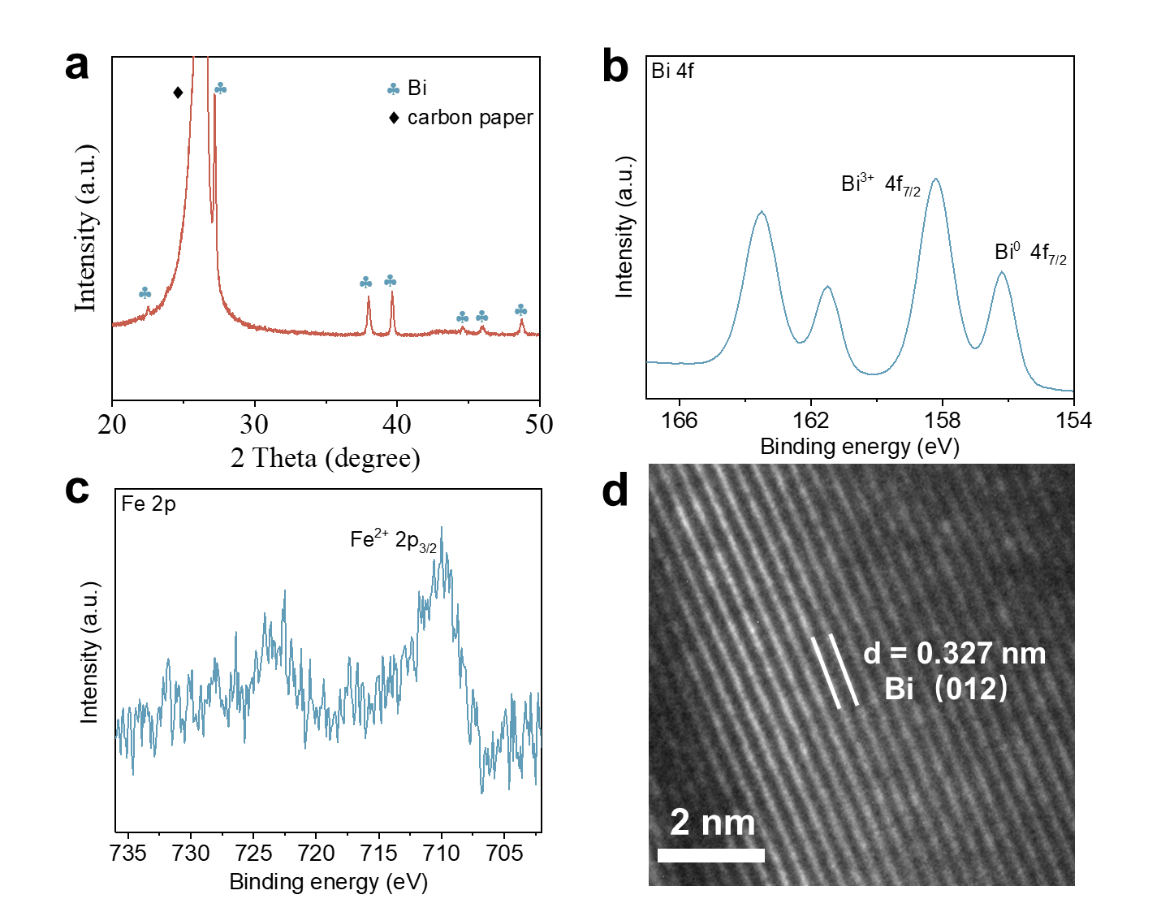


**Figure S27**. (a) XRD pattern of Fe_1_Bi SAA after electrolysis. High resolution (b) Bi 4f and (c) Fe 2p spectra of Fe_1_Bi SAA after electrolysis. (d) HR-TEM image of Fe_1_Bi SAA after electrolysis.


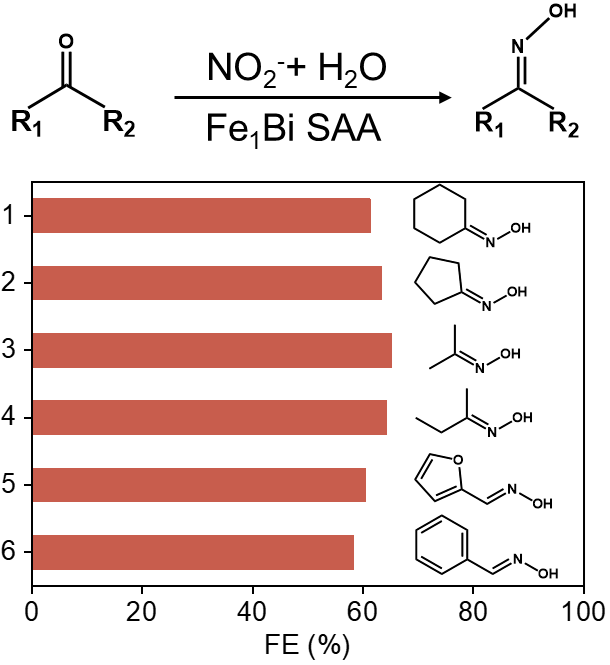


**Figure S28**. The FE of oximes using different ketone or aldehyde substrates and nitrite under the same conditions.


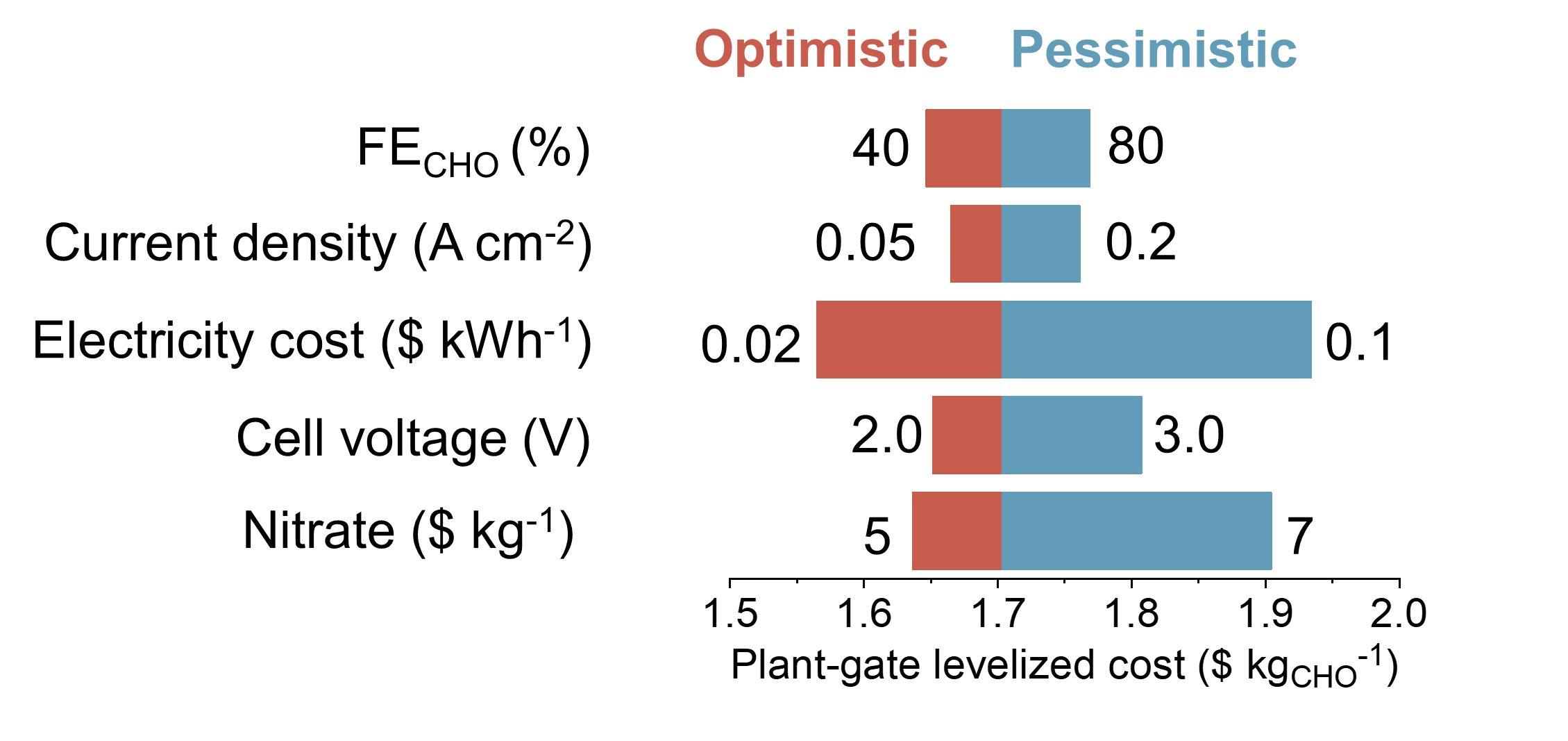


**Figure S29**. Single-variable sensitivity analysis for the production cost of cyclohexanone oxime. The baseline parameters were chosen based on the performance of Fe_1_Bi SAA in the flow electrolyzer, and the values next to the bars indicated each optimistic and pessimistic parameter.


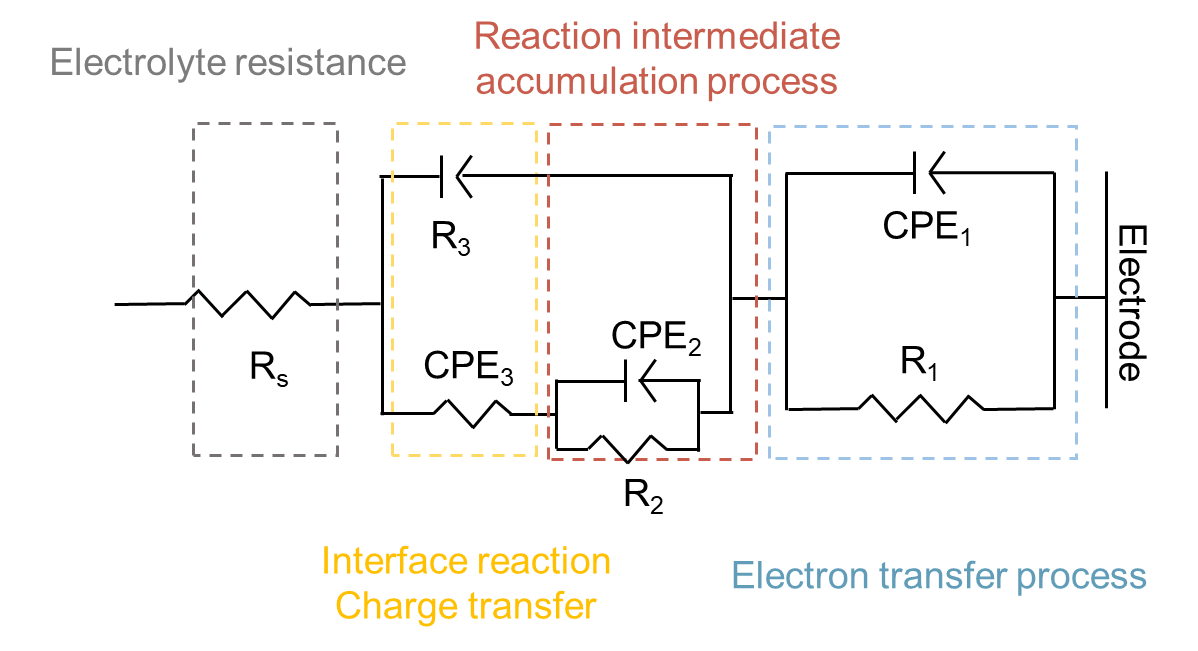


**Figure S30.** The equivalent circuit for Fe_1_Bi SAA.


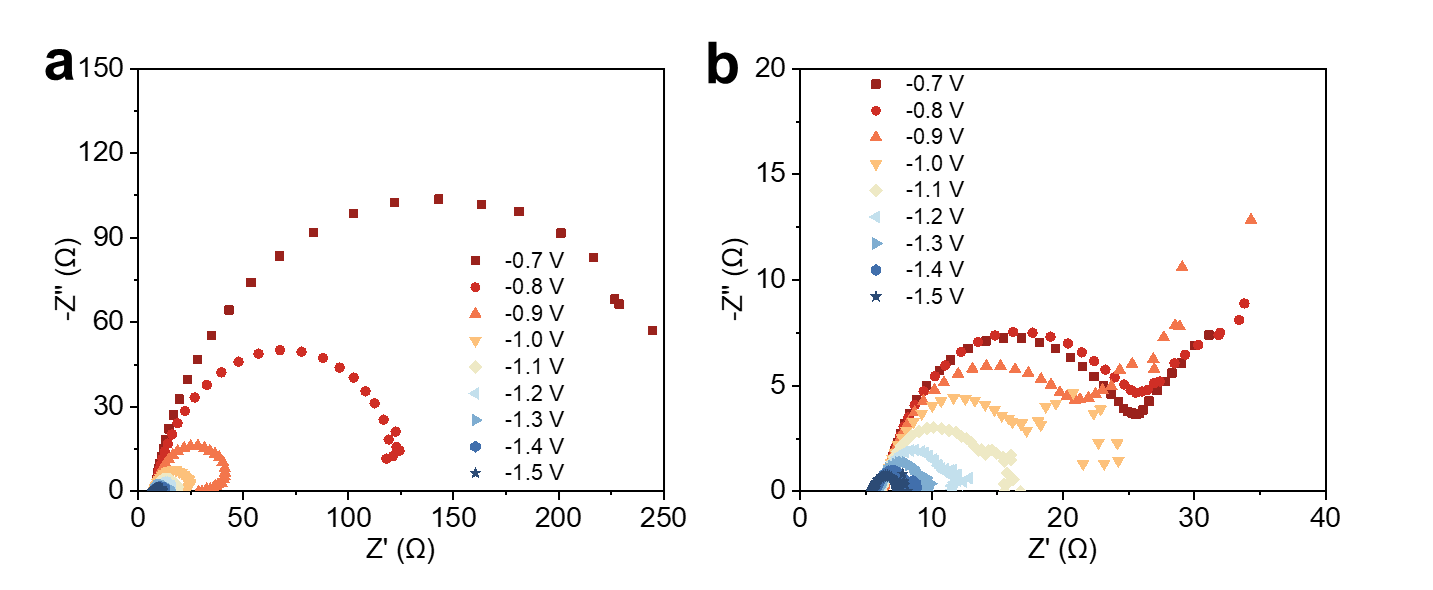


**Figure S31**. Nyquist plots of Fe_1_Bi SAA at different potentials for (a) HER and (b) C-N coupling reaction.

**Figure S32**. CV curve of Fe_1_Bi SAA in PBS.


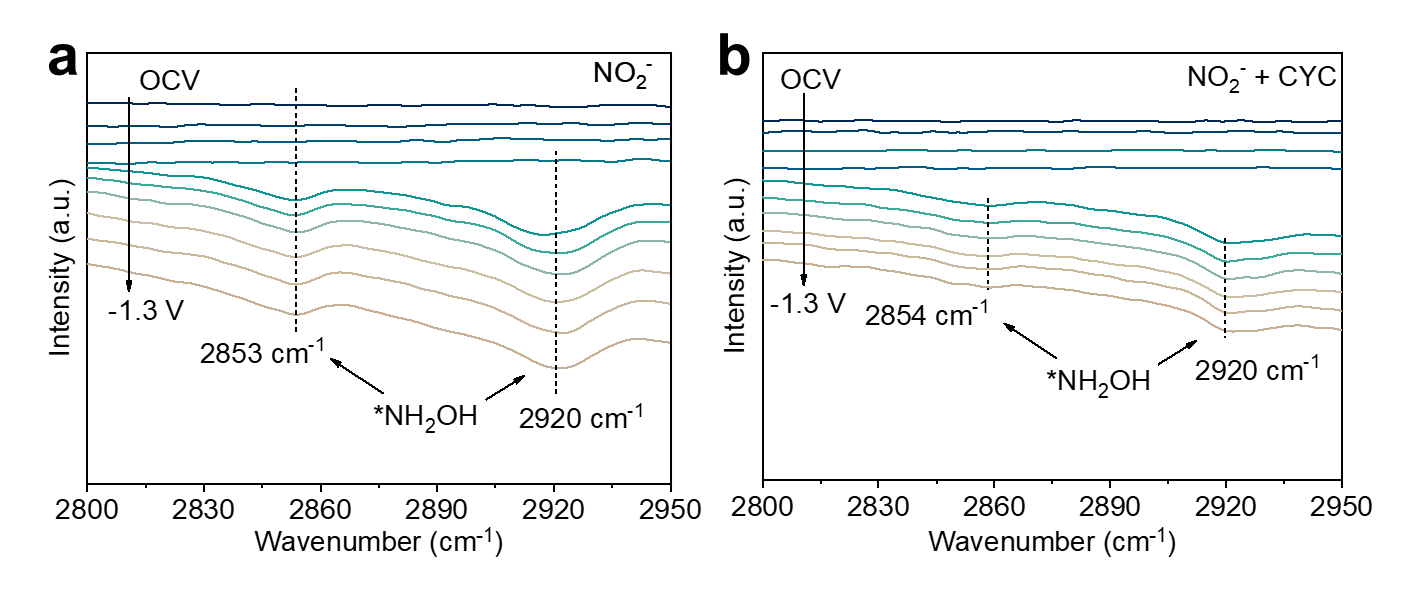


**Figure S33**. In situ ATR-SEIRAS spectra of pristine Bi at different potentials for (a) NO_2_RR and (b) C-N coupling reaction.


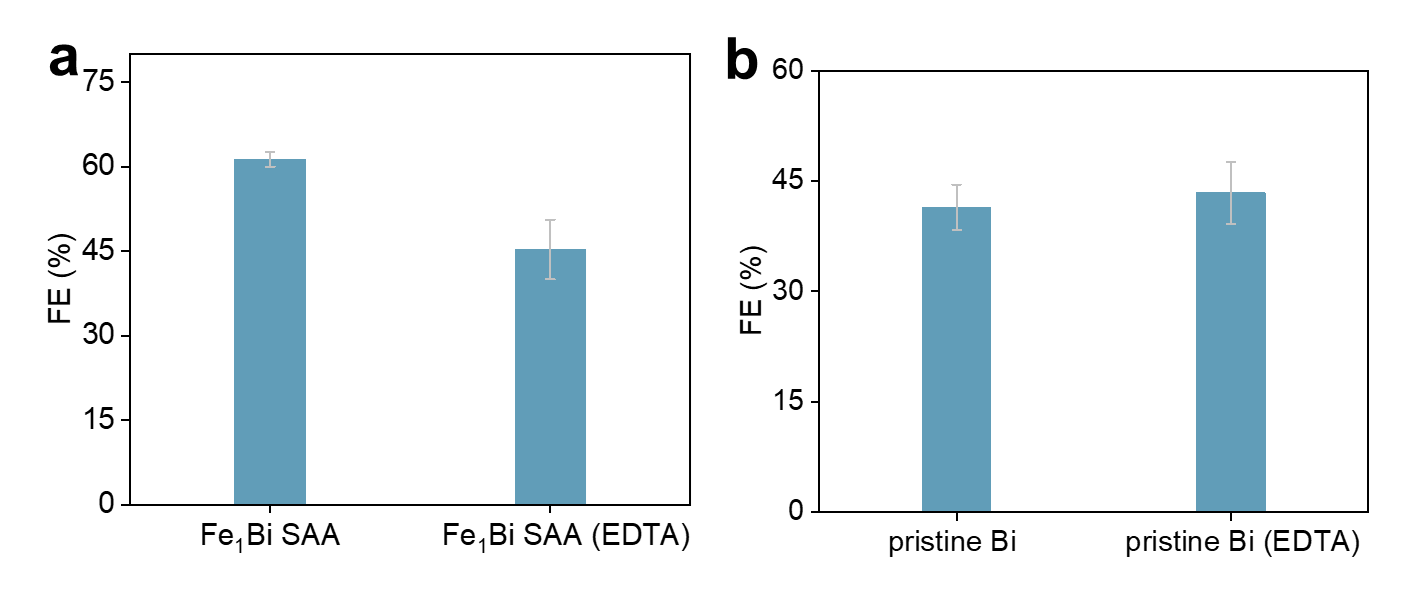


**Figure S34**. Catalyst poisoning experiment for (a) Fe_1_Bi SAA and (b) pristine Bi with the addition of EDTA.

**
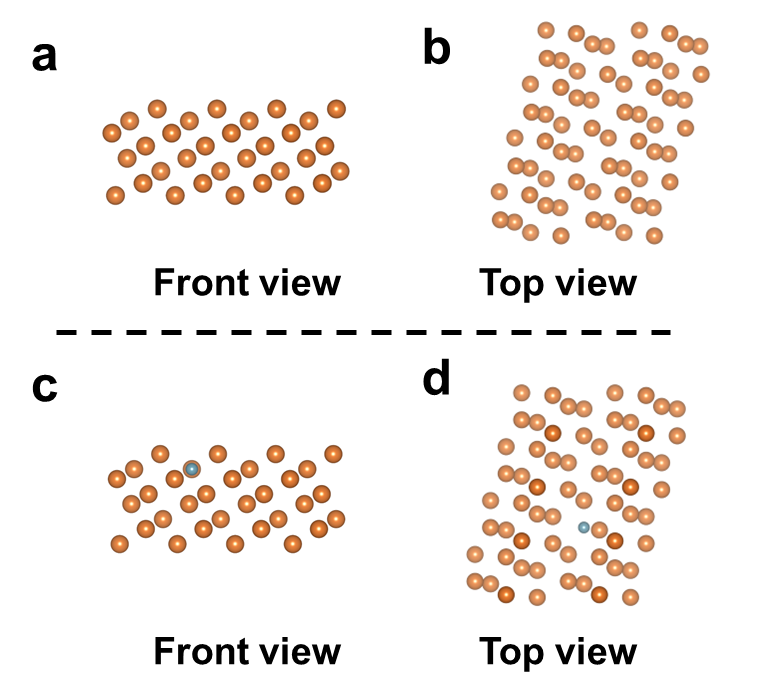
**

**Figure S35**. The optimized structure models of (a, b) pristine Bi and (c, d) Fe_1_Bi SAA.


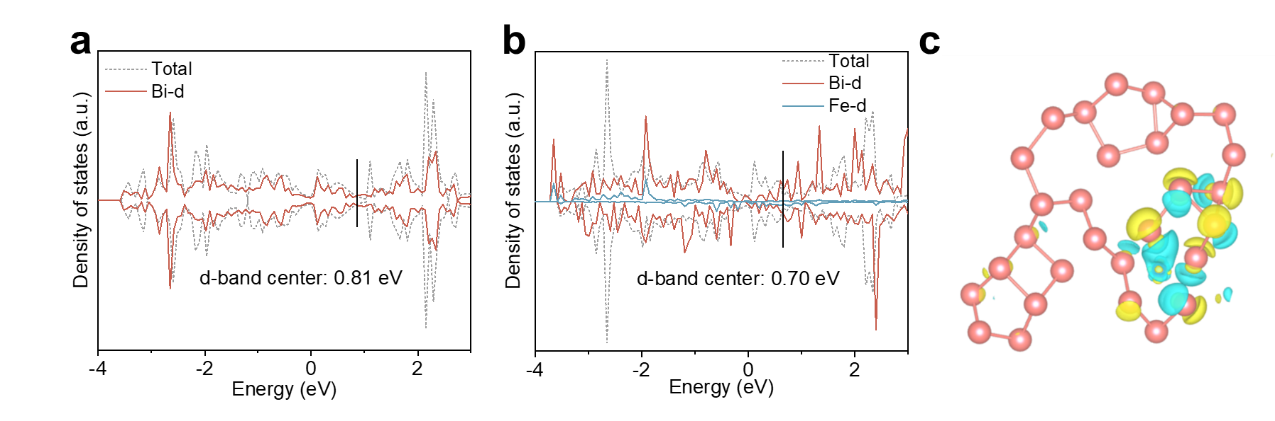


**Figure S36**. Projected density of states for (a) pristine Bi and (b) Fe_1_Bi SAA. (c) Charge density difference of Fe_1_Bi SAA.

**Figure S37**. Gibbs free energy profiles for nitrite reduction on different sites via the intermediate of *NHOH.

**Figure S38**. The Gibbs free energy changes for NO_2_^-^ reduction on pristine Bi.


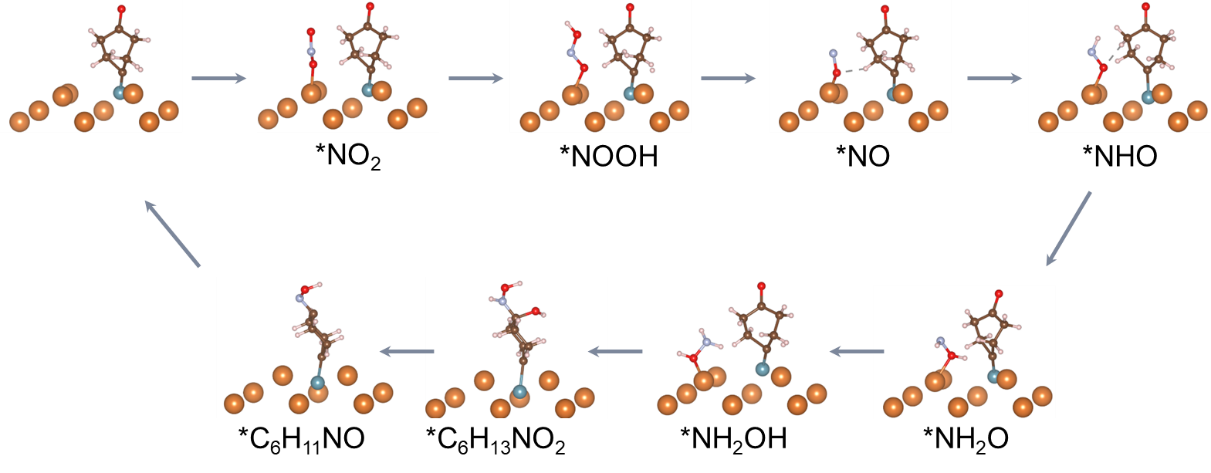


**Figure S39**. Structural configurations of key reaction intermediates over Fe_1_Bi SAA.


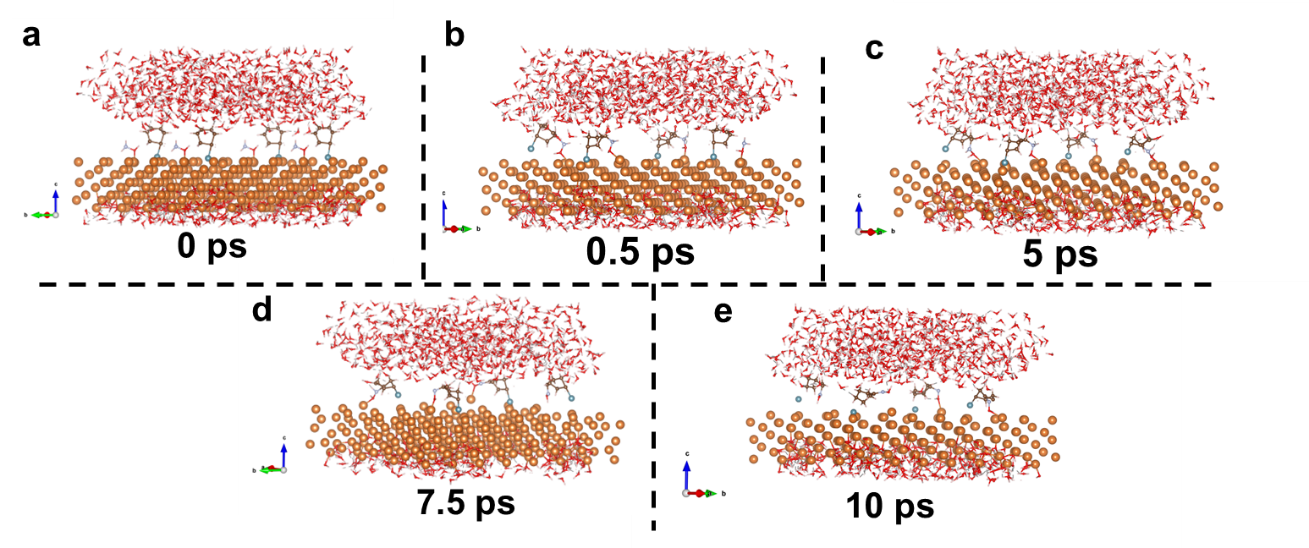


**Figure S40**. (a-e) The snapshots of the MD trajectories of the C-N coupling reaction at 0, 0.5, 5, 7.5, and 10 ps.

**Table S1**. EXAFS fitting parameters at the Fe K-edge (Ѕ_0_^2^=0.98).

| Catalysts | Path | C.N. | R (Å) | σ^2^×10^3^ (Å^2^) | ΔE (eV) | R factor |
| --- | --- | --- | --- | --- | --- | --- |
| Fe_1_Bi SAA | Fe-O | 6.4 | 1.82±0.01 | 2.6±1.9 | -8.30±1.13 | 0.0034 |
|  | Fe-Bi | 4.2 | 2.65±0.01 | 4.1±0.1 | -8.30±1.13 |  |

*C.N.*: coordination numbers; *R*: bond distance; *σ*^2^: Debye-Waller factors; Δ*E*_0_: the inner potential correction. *R* factor: goodness of fit.

**Table S2**. Previously reported catalysts and corresponding electrochemical indicators.

| Catalysts | Cell type | Nitrogen  source | FE | Yield rate  (mmol cm^-2^ h^-1^) | Ref. |
| --- | --- | --- | --- | --- | --- |
| **Fe_1_Bi SAA** | **H cell** | **NO_2_^-^** | **61.3%** | **0.27** | **This work** |
| **Fe_1_Bi SAA** | **MEA (@ 50 mA cm^-2^)** | **NO_2_^-^** | **70.9 %** | **0.33** | **This work** |
| **Fe_1_Bi SAA** | **MEA (@ 200 mA cm^-2^)** | **NO_2_^-^** | **50.2 %** | **0.94** | **This work** |
| Cu-S | H cell | NO_2_^-^ | 26% | 0.165 | 9 |
| Pd_3_Bi | H cell | NO_2_^-^ | 46.1% | 0.50 | 10 |
| HEA-PdCuAgBiIn | H cell | NO_2_^-^ | 47.6% | 0.29 | 11 |
| FeBPAbipyH | H cell | NO_2_^-^ | 77.3% | 0.04 | 12 |
| Zn_93_Cu_7_ | H cell | NO_3_^-^ | 27% | 0.17 | 13 |
| R-TiO_2_ | H cell | NO_3_^-^ | 68.2% | 0.13 | 14 |
| CP/PTFE | H cell | NO | 44.8% | 0.09 | 15 |
| Cu/TiO_2_ | Flow cell | NO_3_^-^ | 51.4% | 0.17 | 16 |
| Cu_1_MoO_x_/NC | Flow cell | NO_3_^-^ | 94.5% | 3.0 mol g^-1^ h^-1^ | 17 |
| Ag-50 | Flow cell | NO_2_^-^ | 83.8% | 0.78 | 18 |
| Ag_16_Cu_18_ | Flow cell | NO | 47.4% | 2.66 | 19 |
| Ag/C | Flow cell | NO | 85.5% | 1.19 | 20 |
| Fe | MEA | NO_3_^-^ | 20% | 55.9 g h^-1^ g_cat_^-1^ | 21 |
| FePc | MEA | NO_2_^-^ | 52.6% | 2.452 | 22 |

**Table S3**. List of control experiments.

| Entry | C source | N source | electricity | product |
| --- | --- | --- | --- | --- |
| 1 | Cyclohexanone | NO_2_^-^ | No | × |
| 2 | - | NO_2_^-^ | Yes | × |
| 3 | Cyclohexanone | - | Yes | × |
| 4 | Cyclohexanone | NH_4_^+^ | Yes | × |
| 5 | Cyclohexanone | NH_2_OH | Yes | √ |
| 6 | Cyclohexanone | NH_2_OH | No | √ |

**Reference**

[1] W. R. Leow, Y. Lum, A. Ozden, Y. Wang, D.-H. Nam, B. Chen, J. Wicks, T.-T. Zhuang, F. Li, D. Sinton, E. H. Sargent, *Science* **2020**, *368*, 1228.

[2] Y. Lum, J. Huang, Z. Wang, M. Luo, D.-H. Nam, W. R. Leow, B. Chen, J. Wicks, Y. Li, Y. Wang, C.-T. Dinh, J. Li, T.-T. Zhuang, F. Li, T.-K. Sham, D. Sinton, E. H. Sargent, *Nat. Catal.* **2020**, *3*, 14.

[3] M. Jouny, J. J. Lv, T. Cheng, B. H. Ko, J. J. Zhu, W. A. Goddard, 3rd, F. Jiao, *Nat. Chem.* **2019**, *11*, 846.

[4] W. Jia, Z. Cao, L. Wang, J. Fu, X. Chi, W. Gao, L.-W. Wang, *Comput. Phys. Commun.* **2013**, *184*, 9.

[5] J. P. Perdew, K. Burke, M. Ernzerhof, *Phys. Rev. Lett*. **1996**, *77*, 3865.

[6] H. Peng, J. P. Perdew, *Phys. Rev. B* **2017**, *95*, 081105.

[7] G. Kresse, J. Furthmüller, *Phys. Rev. B* **1996**, *54*, 11169.

[8] M. Dion, H. Rydberg, E. Schroder, D. C. Langreth, B. I. Lundqvist, *Phys. Rev. Lett*. **2004**, *92*, 246401.

[9] Y. Wu, J. Zhao, C. Wang, T. Li, B. H. Zhao, Z. Song, C. Liu, B. Zhang, *Nat. Commun.* **2023**, *14*, 3057.

[10] Y. Xu, J. Xie, Y. Sheng, H. Yu, K. Deng, Z. Wang, J. Wang, H. Wang, L. Wang, *ACS Catal.* **2025**, *15*, 6606.

[11] Y. Sheng, J. Xie, R. Yang, H. Yu, K. Deng, J. Wang, H. Wang, L. Wang, Y. Xu, *Angew. Chem. Int. Ed.* **2024**, 63, e202410442.

[12] C. Zhang, S. Meng, Y. Jing, C. Wang, X. Zhang, H. Wang, C. Tung, L. Wu, *Angew. Chem. Int. Ed.* **2025**, *64*, e202506546.

[13] J. Sharp, A. Ciotti, H. Andrews, S. R. Udayasurian, S. M. Garcia-Melchor, T. Li, *ACS Catal.* **2024**, *14*, 3287.

[14] L. Luo, L. Li, L. Xu, Y. Yan, S. Zhang, H. Zhou, Z. Li, M. Shao, X. Duan, *CCS Chemistry* **2025,** *7*, 266.

[15] X. Zhang, H. Jing, S. Chen, B. Liu, L. Yu, J. Xiao, D. Deng, *Chem Catal.* **2022**, *2*, 1807.

[16] S. Jia, X. Tan, L. Wu, X. Ma, L. Zhang, J. Feng, L. Xu, X. Song, Q. Zhu, X. Kang, X. Sun, B. Han, *Chem. Sci.* **2024**, *14*, 13198.

[17] R. Zhao, Y. Wang, J. Fu, F. Zhang, L. Wen, Y. Zhao, B. Guan, B. Han, Z. Liu, *J. Am. Chem. Soc.* **2024**, *146*, 27956.

[18] F. Zhang, Q.-Y. Fan, Y.-C. Huang, H. Li, H. Zou, Y. Li, Y. Zou, S. Wang, C. Yang, Y. Lu, H. Yang, *Nat. Synth.* **2025**, *4*, 479.

[19] L. Qin, Y. Chen, Z. Liu, M. Chen, Q. Tang, Z. Tang, *J. Am. Chem. Soc.* **2025**, *147*, 18003.

[20] X. Liu, C. Cheng, J. Zhao, B. Zhang, Y. Wu, *Angew. Chem. Int. Ed.* **2025***, 64,* e202511865*.*

[21] Y. Wu, W. Chen, Y. Jiang, Y. Xu, B. Zhou, L. Xu, C. Xie, M. Yang, M. Qiu, D. Wang, Q. Liu, Q. Liu, S. Wang, Y. Zou, *Angew. Chem. Int. Ed.* **2023**, *62*, e202305491.

[22] J. Li, X. Liu, S.-M. Xu, M. Xu, Y. Wang, Y. Lyu, A.-Z. Li, Y. Wang, X. Wang, T. Zhou, H. Zhou, Y. Peng, X. Li, L. Zheng, H. Duan, *Nat. Synth*. **2025**, *4*, 1598.
